# Deciphering the Nature of *Trp*73 Isoforms in Mouse Embryonic Stem Cell Models: Generation of Isoform-Specific Deficient Cell Lines Using the CRISPR/Cas9 Gene Editing System

**DOI:** 10.3390/cancers13133182

**Published:** 2021-06-25

**Authors:** Lorena López-Ferreras, Nicole Martínez-García, Laura Maeso-Alonso, Marta Martín-López, Ángela Díez-Matilla, Javier Villoch-Fernandez, Hugo Alonso-Olivares, Margarita M. Marques, Maria C. Marin

**Affiliations:** 1 Instituto de Biomedicina (IBIOMED), Universidad de León, 24071 León, Spain; llopf@unileon.es (L.L.-F.); nmartg05@estudiantes.unileon.es (N.M.-G.); lmaea@unileon.es (L.M.-A.); marta.martin@unileon.es (M.M.-L.); adiem@unileon.es (Á.D.-M.); jvilf@unileon.es (J.V.-F.); halono00@estudiantes.unileon.es (H.A.-O.); 2 Departamento de Biología Molecular, Universidad de León, 24071 León, Spain; 3 Departamento de Producción Animal, Universidad de León, 24071 León, Spain; 4 Biomar Microbial Technologies, Parque Tecnológico de León, Armunia, 24009 León, Spain; 5 Instituto de Desarrollo Ganadero y Sanidad Animal (INDEGSAL), Universidad de León, 24071 León, Spain

**Keywords:** p73 isoforms, TAp73, DNp73, mouse embryonic stem cells (mESC), gene editing, pluripotency, differentiation, cell fate, self-renewal, CRISPR/Cas9

## Abstract

**Simple Summary:**

The *Trp73* gene is involved in the regulation of multiple biological processes such as response to stress, differentiation and tissue architecture. This gene gives rise to structurally different N and C-terminal isoforms which lead to differences in its biological activity in a cell type dependent manner. However, there is a current lack of physiological models to study these isoforms. The aim of this study was to generate specific p73-isoform-deficient mouse embryonic stem cell lines using the CRISPR/Cas9 system. Their special features, self-renewal and pluripotency, make embryonic stem cells a useful research tool that allows the generation of cells from any of the three germ layers carrying specific inactivation of p73-isoforms. Characterization of the generated cell lines indicates that while the individual elimination of TA- or DN-p73 isoform is compatible with pluripotency, it results in alterations of the transcriptional profiles and the pluripotent state of the embryonic stem cells in an isoform-specific manner.

**Abstract:**

The p53 family has been widely studied for its role in various physiological and pathological processes. Imbalance of p53 family proteins may contribute to developmental abnormalities and pathologies in humans. This family exerts its functions through a profusion of isoforms that are generated by different promoter usage and alternative splicing in a cell type dependent manner. In particular, the *Trp73* gene gives rise to TA and DN-p73 isoforms that confer p73 a dual nature. The biological relevance of p73 does not only rely on its tumor suppression effects, but on its pivotal role in several developmental processes. Therefore, the generation of cellular models that allow the study of the individual isoforms in a physiological context is of great biomedical relevance. We generated specific TA and DN-p73-deficient mouse embryonic stem cell lines using the CRISPR/Cas9 gene editing system and validated them as physiological bona fide p73-isoform knockout models. Global gene expression analysis revealed isoform-specific alterations of distinctive transcriptional networks. Elimination of TA or DN-p73 is compatible with pluripotency but prompts naïve pluripotent stem cell transition into the primed state, compromising adequate lineage differentiation, thus suggesting that differential expression of p73 isoforms acts as a rheostat during early cell fate determination.

## 1. Introduction

Cells in multicellular organisms continuously sense and respond to environmental changes and internal signals through a complex cascade of regulatory networks required for development and homeostasis. The p53 family, constituted by the p53, p63 and p73 transcription factors, is a central node in the molecular network that integrates information from various pathways in response to physiological and pathological conditions and determines cellular choices between proliferation, DNA replication/repair, differentiation, senescence or apoptosis [1,2]. Due to their shared and evolutionary conserved structure, p73 and p63 partake in many functional properties with p53, but also claim distinct and unique biological roles. Among them, p73 is intertwined with fundamental roles in governing different aspects of cell physiology and development. These include vascular development and angiogenesis, neural progenitor cell stemness and brain neurogenic niche maintenance, cellular metabolism and somatic cell reprogramming, as well as regulation of ciliogenesis and planar cell polarity [3,4,5,6,7,8,9,10,11,12,13,14,15,16,17,18].

The p53 family members have a conserved dual gene structure that encodes for multiple proteins containing different domains, denominated isoforms [19]. Within the family, the *Trp73* gene generates the largest number of isoforms by alternative promoter usage and RNA splicing [19,20]. This gene has two promoters: promoter P1 (upstream of exon 1), from which transactivating-competent isoforms (TA) are transcribed, and promoter P2 (located in intron 3) that generates the truncated dominant negative (DN) isoforms. TAp73 proteins can perform canonical p53-like functions by transactivating p53 targets, inducing cell cycle arrest, apoptosis, senescence and differentiation, but can also regulate a significant portion of non-p53 related genes involved in development and other cell growth-associated functions [21]. On the other hand, the transactivation-defective DNp73 isoforms can act as repressors of the p53 and TAp73 activities by competing for DNA binding to the p53-responsive elements or by forming inactive oligomers [22]. Besides this p53/TAp73 dominant-negative function, DNp73 isoforms also carry out their own distinct activities [23,24] and can endow cells with gain-of function properties [20]. Furthermore, alternative splicing can occur at the 3′-end of the *TP73* gene as well as at the 5′-end of P1-promoter [19]. The carboxy-terminus of this gene is considered as a negative transcriptional regulator containing an additional TA domain, a sterile alpha motif (SAM) involved in protein-protein interaction and a transcriptional inhibitory domain [25]. Thus, all this differential splicing activity generates, in a context-dependent manner, multiple p73 isoforms that differ in their transactivation efficiency and target gene specificity.

The structural complexity, together with the ability of some isoforms to interact and regulate each other [26], makes the study of this gene extremely complicated. Several mouse models have been generated to investigate the functions of p73 and its different isoforms in a physiological context. The first total *Trp73* knockout (p73KO) mouse was produced by gene-targeting in mouse embryonic stem cells (mESC) replacing exons 5 and 6, which encode the DNA binding domain; therefore, it lacks all the functional isoforms [27]. Later, selective knockouts were generated to elucidate the role of each specific isoforms (TA-, DNp73). TAp73 null mice were obtained by deleting exons 2 and 3 of the *Trp73* gene [28], whereas DNp73-deficient mice specifically lack exon 3′ [29,30]. Finally, a selective knockout of the C-terminus of the full-length alpha isoform has been generated switching the TAp73-alpha isoform for the TAp73-beta, but maintaining its physiological expression [31]. While these mouse models have been of critical importance for highlighting the role of p73 in oncogenesis and developmental processes, the identification of the isoform-specific roles has been difficult. The p73KO mice exhibit a myriad of phenotypes with many severe developmental defects, revealing the central role of p73 in development [32,33]. Meanwhile, the phenotypes of the isoform-specific knockouts are more subtle and some of the p73KOs phenotypes do not appear in any of them, probably reflecting either compensatory mechanisms in the absence of one of the isoforms, synergistic relationships between the isoforms and/or possible differences in the genetic background of the mice models [34]. Thus, the elucidation of the molecular pathways specifically regulated by the different p73 isoforms require fine-tuned cellular models sharing the same genetic background and capable of recapitulating p73-regulated processes in vitro.

ESCs, derived from blastocyst-stage mammalian embryos, are pluripotent and can initiate specific differentiation programs related to the three germ layers in vitro. The pluripotent nature of these cells makes them great tools for dissecting cell lineage development in mammals and a limitless source for producing specialized cells for cell therapy and drug testing. ESCs and induced pluripotent stem cells (iPSC), with similar ESC properties, allow the development of physiological 2D and 3D differentiation models and have become an important resource in Biomedicine and Developmental Biology [35,36]. In this context, iPSCs with total *Trp73* loss (p73KO-iPSC) have been derived from mouse embryonic fibroblasts from p73KO mice, generating the first physiological cell model lacking p73 [15]. The p73KO-iPSCs have a defective epithelial phenotype, alterations in the expression of pluripotency markers and defective BMP pathway activation [15]. These cells were successfully used to generate 2D and 3D models of sprouting angiogenesis to address the role of p73 in vascular morphogenesis and angiogenesis [4,5]. However, the total knockout of the gene impedes pinpointing the individual roles of p73 isoforms in the studied processes. Therefore, the development of physiological models that specifically inactivate each isoform individually, all in the same genetic background, is of paramount interest for the p73 research field.

E14TG2ɑ embryonic stem cells (E14) were derived from strain 129/Ola blastocysts [37] and have been extensively used for the generation of germ-line chimeras. In addition, numerous investigations analyzing gene expression programs of these cells at transcriptional and post-transcriptional levels have been reported [38,39,40], making E14 a well-established ESC parental line. In this study, we describe the generation of novel E14-derived mESC lines in which we specifically inactivated p73-isoforms (E14-TAp73KO and E14-DNp73KO) using the CRISPR/Cas9 system. The genetic modifications, deleting either exons 2 and 3 (E14-TAp73KO) or 3′ (E14-DNp73KO), were intended to mimic those produced in the available knockout mice of either isoform [28,30]. Characterization of these cells lines indicated that the loss of TA or DN-p73 isoform did not compromise mESC pluripotency but resulted in isoform-specific alterations of mESC transcriptional profiles and the pluripotent state. RNA sequencing and differential gene expression analysis confirmed many of the known p73 transcriptional targets, validating the generated cell lines as physiological bona fide p73-isoform-specific knockout models.

## 2. Materials and Methods

### 2.1. Cell Culture

The following mESCs lines were used: (i) parental E14 (E14-WT from now on) [37] were kindly provided by Dr. Jim McWhir (former Researcher at the Roslin Institute, Edinburgh, Scotland, UK); (ii) E14-TAp73KO (3 clones) and (iii) E14-DNp73KO cells (3 clones) were generated during the course of this work as novel lab resources. The cell lines were cultured on 0.1% gelatin-coated plates (10^5^ cells/cm^2^) in Glasgow Minimum Essential Medium (GMEM) supplemented with 10% fetal bovine serum (FBS), 2 mM L-glutamine, 1 mM sodium pyruvate, 1 mM nonessential amino acids, 0.1 mM β-mercaptoethanol and 500 U/mL leukemia inhibitory factor (LIF) (stemness culture conditions, S/L).

### 2.2. Single Guide RNA (sgRNA) Design and Validation

Guide RNAs were designed with the Breaking-Cas freely accessible web-based tool [41]. The following CRISPR RNAs (crRNAs), targeting the genomic regions of interest, were selected:

- Guide 1.1 TAAACCTACAATGACGGCCAGGG (position 34875-34898, strand −, score 94)
- Guide 1.2 GAAGGCTGTGACTTGTCGCCAGG (position 34812-34835, strand −, score 92.6)
- Guide 2.1 CCCGAAGCTCTTCCTACCGCTGG (position 36063-36086, strand −, score 92.9)
- Guide 2.2 TCCACGGGGCCTCAAAATGTTGG (position 36202-36225, strand −, score 91.2)
- Guide 3.1 CCCACTGCATGATCGTCATTGGG (position 44081-44104, strand +, score 97.3)
- Guide 3.2 ACCTCTTTGCAGCGCTCCTAGGG (position 43663-43686, strand −, score 89.8)

To prepare the sgRNA, equimolar concentrations (100 μM) of each crRNA (Cultek-Dharmacon, Madrid, Spain) and the ATTO-550 labelled tracrRNA (IDT, Coralville, IA, USA) were resuspended in nuclease-free duplex buffer (IDT, Coralville, IA, USA), following the manufacturer’s instructions. The mixture was incubated for 5 min at 95 °C and cooled down to 4 °C in a thermocycler (ramp rate 5 °C/min). To assemble the ribonucleoprotein (RNP) complex, the sgRNAs were incubated with the purified Cas9 protein (IDT, Coralville, IA, USA) at room temperature (RT) for 10 min.

Cleavage efficiency was assessed in vitro prior to gene editing experiments. Genomic target regions for the different sgRNAs were amplified by PCR (F1: GAGGCTGTGCTGAGGAAATC; R1: CCCCAGAGCTGTGCTAAGTC; F2: CCTTGGGTCGGAAGAAAAAT; R2: GAACAGAGGCCTTCACTTGC; F3: GGAGAGTGGACAGGTTCTGC; R3: ATGGTAGCCACCACTGGTTC) and gel-purified PCR products were incubated with the RPN complex at 37 °C for 3 h, followed by an inactivation step at 65 °C for 10 min. Cleavage products were resolved on a 2% agarose-TBE gel, using SimplySafe^TM^ (EURx, Gdańsk, Poland) for DNA visualization, and 100 bp or 1 kb ladders (Biotools, Madrid, Spain) as size standards.

### 2.3. Gene Editing of mESC

E14-TAp73KO and E14-DNp73KO cell lines were generated from E14-WT cells using the CRISPR/Cas9 system. Briefly, E14-WT cells in suspension were transfected with the RNP complex using Lipofectamine 3000 (Thermo Fisher Scientific, Waltham, MA, USA). The transfection mix was prepared by combining the RNP complex (5 μg of Cas9, 2.5 μg of sgRNA) with 14 μL of P3000^TM^ Enhancer Reagent and 4 μL of Lipofectamine^®^ 3000 in 60 μL of Opti-MEM^TM^-Reduced Serum Medium (Gibco. Thermo Fisher Scientific, Waltham, MA, USA). After 15 min incubation at RT, the mix was added to 400,000 cells resuspended in 400 μL Opti-MEM, which were then split into two wells of a p12-well plate. The day after, cells were recovered and seeded to obtain transfected pools (TAp73KO approach) or single-cell clones (DNp73KO approach), as detailed below. Since the tracrRNA was labelled with the ATTO-550 fluorescent dye (λex554 nm; λem574 nm), transfection was monitored by fluorescence microscopy using a Leica DMI3000 microscope. For the TAp73 gene editing experiment, a total of 141 pools were screened by PCR using primers spanning the targeted region (TA Fw: GGGTGAGGACCATAGAGGGT and Rv: GTGGTCCAGTAGGGGAGGAT). The best candidates were then subjected to two sequential rounds of limiting dilution to clone the edited cells (198 colonies analyzed in total), and the selected clones were verified by PCR and sequencing. To generate the E14-DNp73KO cell line, transfected cells were seeded at clonal density (5000 cells/p150 dish). Cells showing red fluorescence in their nuclei were identified, and 108 colonies were picked and screened by PCR (DN Fw: TTTTCTCCGGTGGAACGAGG and Rv: CCACCACTGGTTCTGAGACC). To obtain homozygosity, the positive colonies were transfected again with the RNP complex. Subsequently, 89 colonies were genotyped, and the correct candidates were validated by PCR and sequencing analysis, as above.

### 2.4. Gene Expression Analysis

Total RNA from cultured cells was isolated using RNeasy mini Kit (QIAGEN, Hilden, Germany). RNA concentration and quality were determined using a Nanodrop ND-100 (Thermo Fisher scientific, Waltham, MA, USA) prior to cDNA synthesis, which was carried out using the High Capacity RNA-to-cDNA kit (Applied Biosystems, Waltham, MA, USA). Gene expression was quantified by qRT-PCR using FastStart Universal SYBR Green Master (Roche, Basel, Switzerland). Primer sequences for target genes used were as follows: TAp73 Fw: GCACCTACTTTGACCTCCCC and Rv: GCACTGCTGAGCAAATTGAAC; DNp73 Fw: ATGCTTTACGTCGGTGACCC and Rv: GCACTGCTGAGCAAATTGAAC; p53 Fw: GTCACAGCACATGACGGAGG and Rv: TCTTCCAGATGCTCGGGATAC; p63 Fw: CAGATTCAGAACGGCTCCTC and Rv: CCGGGTAATCTGTGTTGGAG; Lama5 Fw: GCGTGTGTTTGACCTACACCA and Fv: GGTCTCGATGAGTTGGGCTG; Ccnd2 Fw: GAGTGGGAACTGGTAGTGTTG and Rv: CGCACAGAGCGATGAAGGT; Cdk6 Fw: GGCGTACCCACAGAAACCATA and Rv: AGGTAAGGGCCATCTGAAAACT; Nanog Fw: CTCATCAATGCCTGCAGTTTTTCA and Rv: CTCCTCAGGGCCCTTGTCAGC; Esrrb Fw: GCACCTGGGCTCTAGTTGC and Rv: TACAGTCCTCGTAGCTCTTGC; Zic3 Fw: TGCTGCCAGTTCAGGCTATG and Rv: GCAGAAGGGGTTTTAGTGGTATC; Pou3f1 Fw: TCGAGGTGGGTGTCAAAGG and Rv: GGCGCATAAACGTCGTCCA; 18S (housekeeping gene) Fw: AGTTCCAGCACATTTTGCGAG and Rv: TCATCCTCCGTGAGTTCTCCA. The comparative threshold cycle method was used to quantify relative mRNA expression. To induce *Trp73* expression, cells seeded at a cell density of 77,000 cells/cm^2^ were treated with 0.3 μM of doxorubicin (Sigma-Aldrich, St. Louis, MO, USA) for 12 h [42] prior to RNA isolation.

### 2.5. 2D-Diferentiation Assay

Embryoid bodies (EBs) were prepared by the hanging drop method by placing 20 µL-cell suspension drops (30,000 cells/mL in standard culture medium without LIF, differentiation medium) on the lid of a Petri dish. Three days later, EBs were collected and kept in suspension, in the same medium, for four additional days. EBs were then transferred to 0.1% gelatin-coated tissue culture dishes in Dulbecco’s Modified Eagle Medium (DMEM), supplemented with 20% FBS, 1 mM L-glutamine, 1 mM nonessential amino acids and 0.1 mM β-mercaptoethanol. The medium was changed every other day until day 14, when cells where fixed with 4% paraformaldehyde (PFA) and used for further immunofluorescence analysis (see below). EB images (day 3) were captured with a Nikon SMZ1500 stereomicroscope coupled to a Nikon DS-Fi3 digital camera. Phase-contrast microscopy images along the differentiation process (days 8–12) were obtained using a Leica DMI3000 B microscope coupled to a Leica DFC310 FX digital camera.

### 2.6. RNA Sequencing and Transcriptome Data Analysis

Total RNA from cells of the different genotypes under S/L conditions (day 0) and after three days of EBs-differentiation conditions (day 3) was isolated as described above. Samples from three replicate experiments were sent for RNA-sequencing (RNA-seq) and further bioinformatics analysis to Novogene (UK) Company Limited. The library preparations were sequenced on an Illumina platform and paired-end reads were generated. Clean reads were mapped to the reference genome using the HISAT2 software. Differential expression analysis between groups was performed using the DESeq2 R package. The resulting p-values were adjusted using the Benjamini and Hochberg’s approach for controlling the False Discovery Rate (FDR). Genes with an adjusted *p*-value < 0.05 were assigned as differentially expressed. Gene Ontology (GO) enrichment analysis of differentially expressed genes was performed with DAVID (Database for Annotation Visualization and Integrated Discovery) bioinformatics resources [43]. Venn diagrams were created using Venny 2.1.0. (https://bioinfogp.cnb.csic.es/tools/venny/index.html; accessed on 1 March 2021).

### 2.7. Cell Growth Kinetics and Cell Cycle Analysis

For daily growth curves, cells were seeded at a density of 92,000 cells/cm^2^ and viable cells were counted daily using Trypan Blue dye exclusion. For cumulative growth curves, cell lines were serially passaged every three days by reseeding 550,000 cells in 6-well plates, and the cumulative population doubling (PD) was calculated by adding the PD obtained at each successive passage.

Cells for cell cycle analysis were harvested 24 h after seeding, fixed in 70% ethanol (−20 °C overnight), and stained with 50 μg/mL propidium iodide in phosphate buffer saline (PBS)/0.1% sodium citrate supplemented with 80 μg/mL RNase A at 37 °C in the dark for 1 h. Cells were then subjected to FACS analysis (MACSQuant^®^ Analyzer 10 cytometer, Miltenyi Biotec, Bergisch Gladbach, North Rhine-Westphalia, Germany). The percentage of cells in the different phases of the cell cycle was determined using Flowlogic software (Miltenyi Biotec, Bergisch Gladbach, North Rhine-Westphalia, Germany).

### 2.8. Immunofluorescence

Cells cultured on coverslips for four days at colony-forming density (2,500 cell/cm^2)^ were fixed with 3.7% PFA and permeabilized with PBS/0.5% TritonX-100 (15 min at RT for each step). Cells were then blocked with PBS/10% Normal Goat Serum (NGS) and incubation with primary antibodies was carried out overnight at 4 °C. The following antibodies were used: purified mouse anti-β-catenin (1:200, #610153, BD, Franklin Lakes, ); rabbit polyclonal anti-E-cadherin (1:200, #3195, Cell Signaling, Danvers, MA, USA); mouse monoclonal (F-7) anti-HA-Tag (1:200, sc-7392, Santa Cruz Biotechnology, Dallas, TX, USA); rabbit polyclonal anti-Nanog (1:1000, #AB5731, Sigma-Aldrich, St. Louis, MO, USA); mouse monoclonal (C-10) anti-Oct-3/4 (1:200, sc-5279, Santa Cruz Biotechnology, Dallas, TX, USA); mouse monoclonal (MC-480) anti-SSEA1 (1:100, #MA1-022, ThermoFisher Scientific, Waltham, MA, USA); purified mouse anti-Tubulin β3 (TUJ-1) antibody (1:1000, #MMS-435P, Covance, Princeton, NJ, USA); purified rat anti-mouse CD31 (1:200, #553370, BD, Franklin Lakes, NJ, USA); rabbit polyclonal anti-alpha smooth muscle Actin antibody (1:200, ab5694, Abcam, Cambridge, United Kingdom,); rabbit polyclonal anti-liver FABP antibody (1:250, ab190958, Abcam, Cambridge, United Kingdom,); mouse monoclonal (A60) anti-NeuN (1:100, #MAB377, Millipore, Burlington, MA, USA); rabbit polyclonal anti-Doublecortin (1:200, #4604S, Cell Signaling, Danvers, MA, USA). Cells were then washed and incubated for 1 h at RT with fluorophore-conjugated secondary antibodies (ThermoFisher Scientific, Waltham, MA, USA, #A21242, #A21042, #A21125, #A21206, #A11012, #A21208, #A21241; 1:1000). Nuclei were counterstained with DAPI. Preparations were mounted on glass slides with Fluoromont-GTM Slide Mounting Medium (Electron Microscopy Sciences, Hatfield, PA, USA). Fluorescence was visualized with a Zeiss LSM 800 confocal microscope. Z-stacks were acquired at different magnifications and, when necessary, deconvolution was performed using the nearest-neighbor approach, followed by orthogonal projection at maximum settings (ZEN 3.2).

For the quantification of the colony thickness, cells from the three genotypes were seeded on coverslips at colony-forming density and after four days, the cells were fixed, immunostained with the indicated antibodies and analyzed. Orthogonal views of the Z-stack image analysis were performed with ZEN blue software (ZEN 3.2) obtaining the average of colony thickness.

For the recuperation of the phenotype assays, E14-TAp73KO cells were seeded (20,000 cells/cm^2^) on coverslips and transfected 24 h later using Lipofectamine 3000 (Thermo Fisher Scientific, Waltham, MA, USA) with either pcDNA3.1 empty or containing HA-TAp73. After 24 h in culture, the transfected cells were fixed, immunostained and analyzed as described above.

### 2.9. Immunoblotting

Cells were lysed in lysis buffer (50 mM Tris pH 8, 120 mM NaCl, 0.5% NP-40) supplemented with 2% protease inhibitors (Aprotinin, Leupeptin 20 μg/mL, sodium orthovanadate 1 mM and PMSF 0.1 mg/mL, all from Sigma-Aldrich, St. Louis, MO, USA). Proteins were separated by SDS-PAGE under reducing conditions and transferred onto nitrocellulose membranes using a Bio-Rad wet transfer system. The membranes were then blocked with 5% nonfat dry milk and 1% NGS in TBS-T (Tris buffer saline–0.05% Tween), followed by overnight incubation at 4 °C with the corresponding specific primary antibodies: rabbit polyclonal anti-Nanog (1:2000, #AB5731, Sigma-Aldrich, St. Louis, MO, USA); mouse monoclonal (C-10) anti-Oct-3/4 (1:1000, sc-5279 Santa Cruz Biotechnology, Dallas, TX, USA); mouse monoclonal (6C5) anti-GAPDH (loading control; 1:50,000, ab8245, Abcam, Cambridge, United Kingdom,). After incubation, membranes were washed in TBS-T and HRP-conjugated secondary antibodies were added for 45 min at RT (ThermoFisher Scientific, Waltham, MA, USA #A31460 and #A31430, 1:10,000). HRP-conjugated proteins were detected with Super Signal West-Pico Chemiluminescent Substrate (Pierce, ThermoFisher Scientific, Waltham, MA, USA).

### 2.10. Alkaline Phosphatase (AP) Staining

Cells were seeded at 80 cells/cm^2^ and 24 h later media was changed to either ESC medium without LIF or standard culture ESC medium. After four days, cells were stained for AP activity with NBT/BCIP (5-bromo-4-chloro-3-indolyl-phosphate/nitro blue tetrazolium, Sigma-Aldrich, St. Louis, MO, USA) according to manufacturer’s instructions. Images were obtained with a Nikon EclipseTE300 microscope coupled to a Nikon DXM1200F digital camera.

### 2.11. Statistical Analysis

GraphPad Prism 9.0 (GraphPad Software, San Diego, CA, USA) was used for statistical analyses. All data are presented as mean ± standard error of the mean (SEM). To test for normal distribution, D’Agostino and Pearson tests were performed, and nonparametric assumptions were made when necessary. In that case, the means of two experimental groups were compared with the Mann-Whitney test. Otherwise, unpaired two-tailed Student’s *t*-test for comparison of two groups or two-way ANOVA with post hoc Holm-Sidak’s multiple comparison test was applied when appropriate. *p*-values lower than 0.05 were considered statistically significant. Asterisks denote statistical differences between wild type and genetically modified cell lines (* *p* < 0.05, ** *p* < 0.01, *** *p* < 0.001), while hash symbols were used for comparisons within the same genotype.

## 3. Results and Discussion

### 3.1. Generation of TA- and DN-p73-Specific Knockout mESC by CRISPR/Cas9 Gene Editing

**Figure 1:**
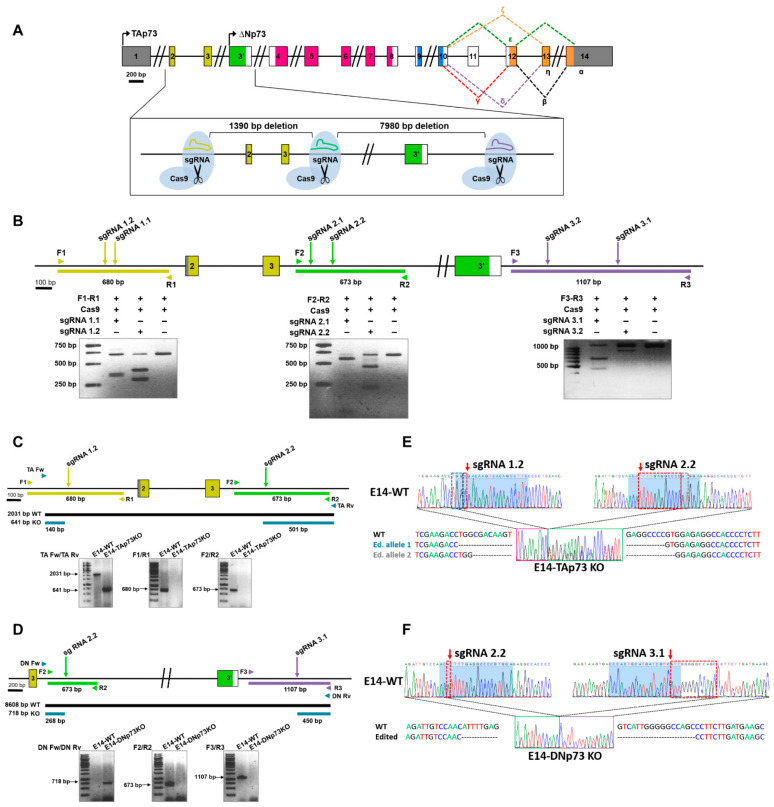
Generation of TA- and DN-p73-specific knockout mESC by CRISPR/Cas9 gene editing. (**A**) Diagram of the followed strategy. (**B**) In vitro assessment of the CRISPR/Cas9-mediated cleavage. Target regions were amplified by PCR from E14TG2α genomic DNA and incubated with the Cas9 protein and the corresponding sgRNA. Cleavage locations are indicated by arrows. (**C**,**D**). The scheme indicates the expected PCR fragments (Primers Fw/Rv) to confirm correct gene editing of the E14-TA- (**C**) and E14-DN- (**D**) p73KO clones. Amplification across the boundaries of the sgRNA binding regions (Primers F1/R1, F2/R2 and F3/R3) showed the expected amplicon size for E14-WT genomic DNA, while no products were detected for the edited clones. (**E**,**F**) Sequencing electropherograms from E14-WT cells and the selected clones (E14-TA and E14-DN-p73KO, respectively). The E14-TAp73KO clone shows a 1390 bp deletion plus small different indels between both alleles in the boundaries of the deletion (**E**), while the E14-DNp73KO clone has a 7890 bp deletion (including a 14 bp indel, red dotted-squares), homozygous for both alleles (**F**). Blue-shadowed sequence corresponds to sgRNA target sequence.

To successfully knockout the *Trp73* gene in mESCs, we decided to reproduce the genetic modifications generated by gene targeting to obtain the TAp73KO and DNp73KO mice [28,29,30]. For this purpose, an editing strategy was designed involving a pair of single guide RNAs as depicted in Figure 1A,B. For TAp73 inactivation, sgRNAs were homologous to the target sequence of the *Trp73* gene upstream of exon 2 (sgRNA1) and downstream of exon 3 (sgRNA 2). DNp73 inactivation was performed using two sgRNAs flanking exon 3′ (sgRNA 2 and sgRNA3 at the 5′ and 3′ ends, respectively). These guides were selected according to two criteria: the highest score, which indicates the best oligo with less predicted off-targets; and the location of the off-targets.

To generate the TA- and DN-p73KO cell lines, E14-WT cells were transfected with the specific ribonucleoprotein complex containing the selected sgRNAs and the Cas9 protein, and transfection was monitored by fluorescence microscopy. For the TAp73 gene editing experiment, a total of 141 pools were screened by PCR and the best candidates were then subjected to two sequential rounds of cloning by limiting dilution, where a total of 198 colonies were screened. To generate the E14-DNp73KO cell lines, the strategy was slightly different, and the transfected cells were seeded at clonal density and fluorescence colonies (108) were picked and screened by PCR. To obtain homozygosity, the selected clones were again transfected with the RNP complex and seeded at low density, from which 89 colonies were picked and genotyped. All the candidate clones for both genotypes were then validated by PCR and sequencing analysis.

Amplification of the expected PCR products in the knockout clones (Figure 1C,D; PCRs with primers “Fw” and “Rv”) confirmed that dual sgRNA-targeted cleavage resulted in the deletion of the intervening region between the two double strand breaks. Diagnostic PCR analysis for TAp73KO (Figure 1C, Supplementary Figure S1A) showed that, whereas wild type control cells (E14-WT) displayed a 2031 pb fragment spanning exons 2 and 3, the amplicon in the E14-TAp73KO cells was of the expected reduced size (641 bp). Similarly, E14-p73DNKO cells showed the expected 718 bp diagnostic fragment (Figure 1D, Supplementary Figure S1B); however, amplification with DN Fw and DN Rv primers (including part of intron 3 and exon 3′) did not work in E14-WT cells, probably due to the large size of the amplicon (8608 pb). In agreement with proper gene editing, no products were detected when amplifying across the boundaries of the sgRNA target regions in the knockout clones, while fragments of the right size were obtained in the wild type (E14-WT) control (Figure 1C,D; PCRs with primers “1”, “2” and “3”). Amplicons spanning the deleted regions were analyzed by Sanger sequencing (Figure 1E,F, Supplementary Figure S1C). Paired Sequence Alignments [44] confirmed that the selected clones carried biallelic deletions of the expected sizes, either 1390 bp (E14-TAp73KO) or 7890 bp (E14-DNp73KO). While the deletion in E14-DNp73KO was homozygous for both alleles (including an additional 14 bp indel; Figure 1F), nonhomologous end-joining repair in the E14-TAp73KO cells produced different indels in both alleles in the boundaries of the Cas9 cleavage site, as shown in Figure 1E.

During the generation of the cell lines, we obtained several CRISPR-edited clones for each knockout genotype. Three lines of each genotype were further analyzed corroborating: (i) the elimination of the targeted isoform, (ii) the expression of the other isoform and p53-family members, and (iii) their morphological and pluripotent phenotype (Supplementary Figures S1–S6). Altogether, these results demonstrated that we successfully generated novel mESC lines with specific inactivation of TA- or DN-p73 isoforms. Furthermore, one clone of each genotype (clone 3 and clone 99 for TAp73 and DN-p73 inactivation respectively, hereafter referred as E14-TAp73KO and E14-DNp73KO lines), was selected to perform a more comprehensive characterization.

### 3.2. Elimination of TA- or DN-p73 Isoforms Induces Isoform-Specific Changes in the mESC Transcriptional Profiles

**Figure 2:**
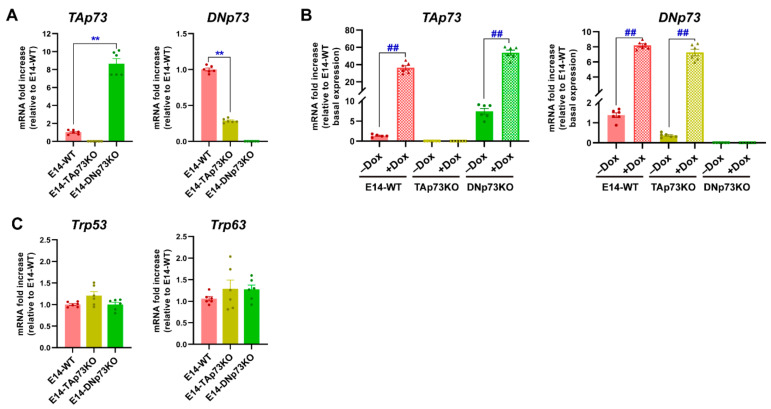
Specific elimination of the TA- or DN-p73 isoform in mESC results in deregulation of the other isoform but does not increase the expression of other family members as part of a compensatory mechanism. (**A**,**B**) qRT-PCR analysis of TA- and DN-p73 expression levels under basal conditions (**A**) or upon treatment with doxorubicin (Dox) (**B**). (**C**) Expression of *Trp53* and *Trp63*, analyzed by qRT-PCR. The data represents the mean ± SEM of three independent experiments. **, ## *p*-value < 0.01.

Once the cell lines were generated, we sought to confirm that the *Trp73* isoform expression correlated to the genotype. Under self-renewal/stemness culture conditions (serum with LIF, S/L), qRT-PCR analysis showed low but detectable levels of TAp73 and DNp73 in E14-WT mESCs, DNp73 being the predominant isoform in accordance with previously published data [5]. However, no sign of TAp73 or DNp73 expression was detected in the chosen E14-TAp73KO or E14-DNp73KO mESCs, respectively, nor in the other analyzed lines (Figure 2A and Supplementary Figure S2A). Nevertheless, we observed a significant deregulation of the remaining isoform when compared to control levels. Whereas DNp73 was downregulated in E14-TAp73KO cells, TAp73 was upregulated in DNp73KO (Figure 2A). The lower DNp73 expression observed in E14-TAp73KO cells coincides with TAp73′s role as a strong transcriptional activator of DNp73 expression [45,46], though there are other factors that can affect DNp73 expression [47,48]. On the other hand, the *Trp73* P1-promoter also contains nonconsensus p53-binding sites [46]; thus, the absence of DNp73, with a dominant negative function could serve as a potentiator of a positive TAp73 feedback loop, resulting in elevated TAp73 levels in E14-DNp73KO cells. Nevertheless, to rule out the possibility that the differences were due to difficulties in detecting the low *Trp73* transcriptional levels, we treated the cells with doxorubicin, a chemotherapeutic agent shown to induce TA- and DN-p73 expression in somatic as well as ESCs [42,49]. This approach resulted in a significant induction of TA- and DN-isoforms (Figure 2B and Supplementary Figure S2B), reaching levels in E14-DN and E14-TA-p73KO cells like those attained in control cells, all confirming that the TAp73KO and DNp73KO mESC models were successfully established. The same trend was observed in the other clones (Supplementary Figure S2B).

Since there is evidence of functional redundancy with other family members, as well as compensatory mechanisms in the p53 family knockout models [1,28,50], we asked whether expression of *Trp53* or *Trp63* would be altered in the isoform-deficient cell lines. The transcription factor p53 is abundantly expressed in ESCs [51,52]. Consistently we found abundant but similar expression of p53 in all cell lines (Figure 2C and Supplementary Figure S2C). On the other hand, previous reports indicate that no DNp63 nor TAp63 expression could be observed in undifferentiated proliferating mESC [53] and, in agreement, we found low but variable expression of p63 in E14-TA- and E14-DN-p73KO cell lines (Figure 2C and Supplementary Figure S2C). Thus, altogether our data indicates that the specific elimination of one of the p73-isoforms in mESC results in a deregulation of the basal levels of the other isoform; however, it does not trigger a significant compensatory upregulation of the other family members.

**Figure 3:**
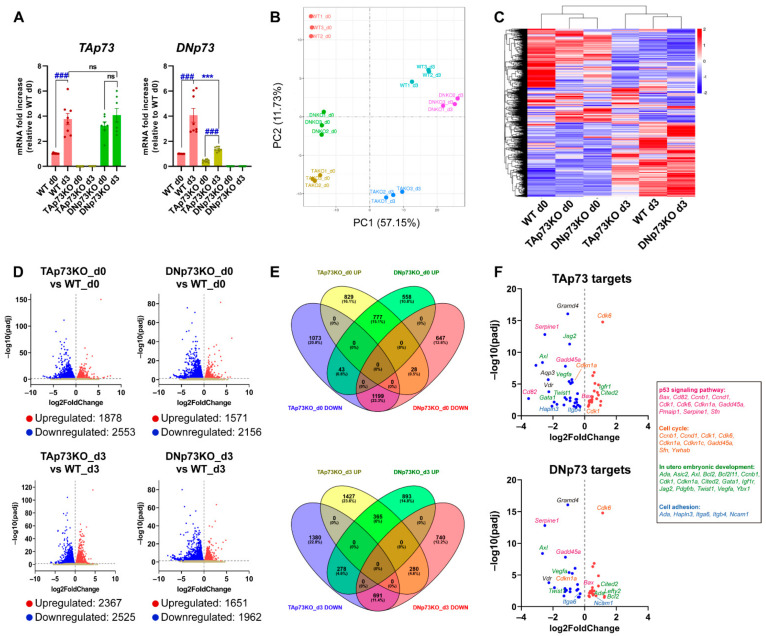
Elimination of the TA- or DN-p73 isoforms results in alteration of mESC transcriptional profiles with some genes showing isoform-specific deregulation, while others are affected by either isoform knockout. (**A**) Expression of p73-isoforms, analyzed by qRT-PCR, under differentiation-permissive conditions (after three days of EBs culture, d3) compared to stemness basal conditions (d0, S/L). At least, three independent experiments were performed. ***, ### *p*-value < 0.001. (**B**) Two-dimensional principal component analysis (2D PCA) score plot showed that samples clearly segregate from each other, demonstrating clear transcriptional differences among groups. (**C**) Hierarchical clustering analysis of expressed transcripts in the different cell lines under either stemness (d0) or differentiation-permissive conditions (d3). (**D**) Volcano plots of differentially expressed genes between E14-TAp73KO and E14-DNp73KO samples vs. E14-WT controls at the different time points analyzed. The estimated log2 Fold Change is plotted against log10 adjusted *p*-value (p-adj). Upregulated and downregulated genes are highlighted as red and blue dots, respectively. Genes that are not differentially expressed are represented as gray dots. (**E**) Venn Diagrams illustrating the relationship of up and down-differentially expressed transcripts in E14-TAp73KO and E14-DNp73KO samples compared to E14-WT controls at day 0 and day 3. The percentage of changed transcripts in each group is shown in parentheses. (**F**) Volcano plots showing differentially expressed genes (DEGs) between knockout cells and WT controls (S/L conditions) that were found to be common to a panel of validated p73 targets associated with p73 canonical and noncanonical functions. Genes associated to GO terms “p53 signaling pathway” (pink), “cell cycle” (orange), “in utero embryonic development” (green) and “cell adhesion” (blue) are indicated.

Next, we asked whether the physiological regulation of the *Trp73* gene remained intact in the generated cell lines. To that purpose, we analyzed p73 expression levels during mESC induced differentiation, since the p53 family members are upregulated during this process [2,53]. Thus, we cultured the generated cell lines under self-renewing culture conditions (S/L, day 0) or under differentiation-permissive EB formation conditions (day 3, serum without LIF) in which mESC recapitulate the signaling and transcriptional events of germ layer specification (reviewed in [54]). TAp73 levels significantly increased in E14-WT cells, and similar levels were reached in all DNp73KO-cell lines after three days of EB culture (Figure 3A and Supplementary Figure S2D). However, despite the reduced DNp73-basal levels in TAp73KO cells (Figure 2A), its expression was significantly increased upon induction of differentiation in all TAp73KO cell lines, (Figure 3A and Supplementary Figure S2E). Nevertheless, the attained DNp73 levels were variable, but generally lower, than those of WT controls (Figure 3A and Supplementary Figure S2E).

To examine the effect of TAp73 versus DNp73 inactivation, we used high-throughput RNA-seq data (Supplementary Table S1) to perform genome-wide transcriptome profiling of the three genotypes under self-renewal or differentiation culture conditions (day 0 and day 3, respectively). Principal component analysis (PCA) indicated that biological replicates clustered closely and revealed a clear separation between the parental and knockout cells on day 0 (Figure 3B). In addition, differentiation conditions were the major driver of variability between the analyzed samples. This was in accordance with previously published data from temporal transcriptomics of mEB differentiation, where the major contributor to the determination of variance was explained by time [55]. Hierarchical clustering (Figure 3C) confirmed that elimination of the individual p73-isoforms influences the transcriptional profiles of mESC, not only under self-renewal conditions, but also in the early stages of differentiation, particularly in the case of E14-TAp73KO, suggesting that TAp73 might be the most relevant isoform regulating early stages of development.

Analysis of the differentially expressed genes (DEGs) at day 0 revealed 1878 upregulated and 2553 downregulated genes in TAp73KO cells compared to E14-WT cells (Figure 3D). Similarly, 3727 DEGs were identified in DNp73KO cells relative to E14-WT cells, with 1571 upregulated genes and 2156 genes being downregulated (Figure 3D), suggesting that, despite the lower levels of *Trp73* in proliferating undifferentiated ESCs, specific elimination of the isoforms resulted in altered transcriptional profiles. Comparison of the transcriptome of TAp73KO and DNp73KO cells with that of WT cells after three days of differentiation revealed 2525 and 1962 differentially downregulated genes, respectively, whereas 2367 and 1651 genes were upregulated in either TAp73KO or DNp73KO cells (Figure 3D).

Next, we further investigated the genes under the direct or indirect transcriptional control of either TA- or DN-p73 (Figure 3E). By pairwise comparisons, we found that these DEGs could be grouped in three categories: (1) genes whose expression was altered in the same way by both isoforms, (2) genes that were specifically affected by the elimination of one isoform, but not the other and (3) genes whose expression was altered by both isoforms but in opposite ways (Supplementary Table S2). Under self-renewal culture conditions (S/L, day 0), inactivation of one or the other isoform produced the same effect in a substantial proportion of genes (category 1: 777 genes (15.1%) commonly upregulated and 1199 genes (23.3%) commonly downregulated), pointing to a core of genes that were transcriptionally controlled in the same direction by both isoforms. In addition, unique isoform-specific transcriptomic signatures were uncovered for each isoform (category 2). In this category, TAp73 loss had the larger impact on gene expression profiles, as suggested by the higher proportion of genes exclusively up or downregulated when TAp73 was inactivated, possibly reflecting direct or indirect TAp73 targets. In the third category, we found 43 genes that were significantly downregulated in E14-TAp73KO and upregulated in E14-DNp73KO. A closer examination of genes with the strongest log2 Fold Change (log2FC) in TAp73KO cells revealed genes such as the neural stem cell marker *Hes3* ([56]; log2FC −5.95), or as Nexilin ([57]; *Nexn*; log2FC −2.63), which encodes an actin filament-binding protein localized at cell-matrix adherens junctions. On the other side, among the 28 genes that were downregulated in E14-DNp73KO (and upregulated in E14-TAp73KO), we found genes such us the Mesenchyme Homeobox 1 (*Meox1*) (log2FC −2.22) or the glutathione peroxidase 2 gene (*Gpx2*; log2FC −1.40). It is of note that these genes have been proposed as p53 family target genes. *Meox1* is mainly expressed during somitogenesis, but has also been linked to cancer, and it is negatively regulated by p53 and PTEN in breast cancer cells [58]. Finally, p63, particularly DNp63, upregulates *Gpx2* [59], which has been proposed as an important player in the modulation of cell fate decisions in the murine intestinal epithelium [60]. Nevertheless, whether these genes are p73 direct transcriptional targets remains to be determined.

Similar comparisons were performed with DEGs obtained under EB-differentiation conditions (day 3, Figure 3E). Regarding DEGs in category 2, Venn diagram representation showed that 1427 (23.6%) and 893 (14.8%) distinct genes were specifically upregulated in E14-TAp73KO or E14-DNp73KO, respectively, and that a similar proportion of genes was specifically downregulated for each isoform (22.8% in TAp73KO-cells and 12.2% in the case of DNp73KO-cells), suggesting altogether that the isoform-specific functions are finely tuned during mESC differentiation. Genes exclusively downregulated in E14-TAp73KO (putative TAp73 target genes), were significantly associated with GO terms related to biological process like “multicellular organism development” (p-adj 4.9 ×10^−3^), “nervous system development” (p-adj 2.7 × 10^−3^) or “cell adhesion” (p-adj 4.9 × 10^−3^), among others. Likewise, functional annotation of downregulated genes in E14-DNp73KO revealed genes involved in “signaling pathways regulating pluripotency of stem cells” (p-adj 4.1 × 10^−3^), “neural tube closure” (p-adj 3.6 × 10^−2^) or “axon guidance” (p-adj 4.1 × 10^−3^), highlighting the specific functions of p73 isoforms and, therefore, the need of a differential regulation of their expression to orchestrate specific developmentally associated phenotypes.

Finally, we sought to test whether the generated cell lines could be a bona fide model to identify and study new p73-isoform-specific targets, as well as to confirm previously reported p73 functions and targets. Recently, Wang et al. (2020) comprehensively evaluated TAp73 transcriptional targets identified in cancer cell-based studies, providing a set of candidate genes derived from both gene-focused and genome-wide studies. When comparing those lists with the DEGs in E14-TAp73KO cells under self-renewal conditions, we found that 25% of the DEGs matched the validated p73 targets (Figure 3F; Supplementary Table S3). Moreover, a similar scenario was applicable to E14-DNp73KO (day 0), in which 20% of the DEGs were known p73 targets. Interestingly, from the 67 published p73-targets that were DEGs in our study, 39 (58%) belong to category 2, suggesting that they are exclusively affected by the elimination of one of the isoforms. GO analysis showed enrichment for pathways like “p53-signaling pathway” (p-adj 2.1 × 10^−11^) or “cell cycle” (p-adj 3.3 × 10^−6^), which include p53 family target genes such as *Cdkn1a*, *Cd82/Kai1*, *Gadd45a*, *Pmaip1*, *Sfn* or *Serpine*, reflecting the p73 canonical functions. Other p73-targets such as *Gata1*, *Itgb4*, Itga6, *Lima1*, *Neurl1a*, *Lefty2*, *Twist1* or *Vegfa* could be linked to p73 noncanonical functions [21,61].

Altogether, these results indicate that the generated cell lines are bona fide physiological models that could be relevant, not only to better understand the role of p73 in the molecular networks controlling cell proliferation and death, but also to decipher other fundamental functions of p73 involved in the regulation of cellular physiology during development, as well as in the adult organism.

### 3.3. TAp73 Is a Regulator Hub of Cell Adhesion Transcriptional Networks

**Figure 4:**
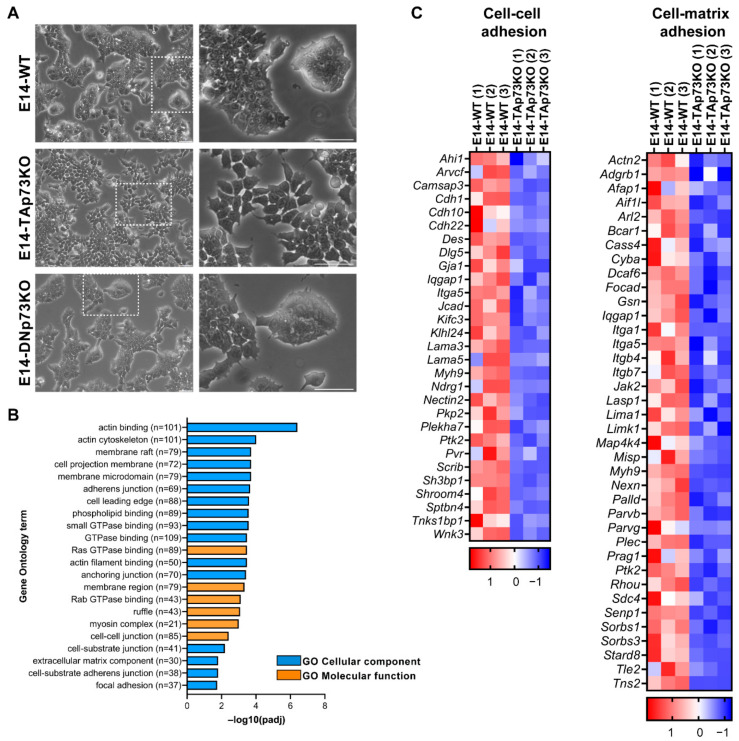
p73 regulates cell adhesion gene networks in mESC. (**A**) Representative images of the three cell lines when seeded under proliferating culture conditions (S/L). The insert represents a magnification from the indicated area (white square). Scale bar: 250 μm. (**B**) Gene Ontology (GO) analysis for downregulated genes in E14-TAp73KO samples compared to E14-WT controls. The number of genes for each term is indicated. (**C**) Heatmaps of down-regulated DEGs in E14-TAp73KO vs. E14-WT under stemness conditions. The cell-cell adhesion heatmap includes the DEGs in common among the GO categories “adherens junction”, “anchoring junction” and “cell-cell junction” from (**B**). Likewise, the cell-matrix adhesion heatmap contains the overlapping genes from the categories “cell-substrate junction” and “focal adhesion”. RNA-Seq expression Z-scores for each gene are plotted.

We previously published that total loss of p73 in iPSC resulted in alterations in the epithelial phenotype of the colonies [15]. To evaluate if the individual elimination of p73-isoforms in mESCs recapitulates this defect, we analyzed the clone’s morphology when seeded under S/L culture conditions. As expected, parental E14-WT cells formed tight domed islands and colonies with refringent borders (Figure 4A, white squares magnification, and Supplementary Figure S3A). Conversely, the TAp73KO cells were scattered, suggesting that TAp73-deficiency hinders the capacity of the cells to establish appropriate intercellular junctions (Figure 4A). Moreover, this phenotype was detected in all the analyzed E14-TAp73KO clones, but not in the DNp73KOs, indicating that the observed phenotype is specific to TAp73-deficient cells and not due to clonal variability generated during the selection process (Supplementary Figure S3A).

TAp73 regulation of epithelial organization has been reported in several models. In ependymal cells, TAp73 regulates cell organization along the plane of the epithelial sheet (Planar Cell Polarity) and modulates, directly and/or indirectly, transcriptional programs regulating actin and microtubules dynamics [18]. A p73-regulated core gene set involved in cell adhesion and cell migration has also been identified in murine ovarian granulosa cells [62]. To gain mechanistic insight into the effect of TAp73 elimination in mESC in tissue architecture, we performed functional annotation of downregulated DEGs in E14-TAp73KO compared to E14-WT cells. GO enrichment analysis not only corroborated previous results on TAp73 modulation of actin dynamics [18] but also identified numerous GO categories related to biological adhesion that were significantly downregulated (p-adj < 0.05) in the absence of TAp73. These include “adherens junction”, “anchoring junctions”, “cell-substrate adhesion”, or “extracellular matrix component” (EMC), among others (Figure 4B). All these transcriptional programs affected by TAp73 elimination relate to typical features of epithelial tissue architecture establishment and maintenance. Among the DEGs involved in “cell-cell adhesion” and “cell-substrate adhesion” (Figure 4C), we identified several cadherin, integrin and laminin coding genes. Some of those downregulated genes in E14-TAp73KO cells have previously been reported as p73 targets like *Itgb4* [63] or *Lama5* [62]. In this regard, we analyzed *Lama5* expression in all the E14-TAp73KO cell lines to confirm that the detected transcriptional changes were common to all of them (Supplementary Figure S3B). Interestingly, our RNA-seq data revealed the downregulation of a set of integrins (*Itga1*, *Itga5*, *Itgb7*), together with focal adhesion kinase FAK (*Ptk1*) suggesting alterations in the cell-EMC interactions in the absence of TAp73. However, it is important to point out that we detected high variability in the levels of the adhesion genes *Itgb4* and *Itga3* in all the cell lines, including WT, among experiments (data not shown), making it difficult to address whether the lack of TAp73 was indeed the underlying cause of the detected differences in gene expression. Thus, further analysis is required to demonstrate p73 involvement in integrin associated-signaling. Altogether, our results in the newly generated cell lines provide additional evidence reinforcing the role of TAp73 as a central hub in the regulation of the epithelial architecture gene networks. These alterations in the adhesome capacity of E14-TAp73KO cells might also hinder their pluripotency state, since the establishment of cell junctions involving E-cadherin is crucial for colony compaction and full pluripotency maintenance [64].

### 3.4. p73-Isoforms Are Expendable for mESC Self-Renewal But Are Important in the Control of G1 Phase, a Permissive Phase for the Initiation of Cell Fate Decision in mESC

**Figure 5:**
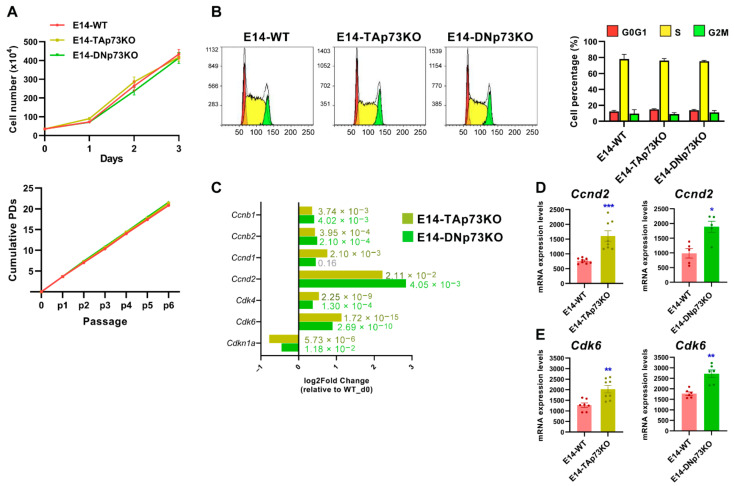
p73-isoforms are dispensable for mESC self-renewal but required for appropriate expression of G1 phase Cyclins/Cdk complexes. (**A**) Short-term and long-term growth curves of the indicated cell lines. Three independent experiments, with three replicates per condition, were performed. (**B**) Representative cell cycle profiles of the control and knockout cell lines. The quantification of the percentage of cells in each cell cycle phase using the Watson improved model showed no differences between genotypes. Bar graphs represent the mean ± SEM from three independent experiments. (**C**) Differential gene expression analysis of cell cycle-related genes (RNA-seq data) in E14-TA- and E14-DN-p73KO cells compared to E14-WT controls at day 0. (**D**,**E**) Analysis of *Ccnd2* and Cdk6 upregulation by qRT-PCR analysis in E14-TAp73KO cells, respectively. * *p*-value < 0.05, ** *p*-value < 0.01, *** *p*-value < 0.001.

The role of p73 in cell proliferation seems to be cell context-dependent. While in iPSCs total *Trp73* deficiency did not affect their proliferative capacity or self-renewal [15], p73 loss in primary embryo fibroblasts resulted in impaired proliferation and premature senescence [65]. Therefore, to assess the impact of the p73 isoform-specific loss on cell cycle regulation and proliferation in mESCs, we compared short and long-term cellular growth kinetics curves from the three genotypes. As depicted in Figure 5A, we did not detect differences in the proliferation rate in any case. Moreover, all the established cell lines were maintained in culture for more than 30 passages without detecting any differences in their population doubling rates. In agreement, cell cycle analysis did not show differences in the percentage of S phase cells among the lines (Figure 5B) and observed cell cycle distribution was in accordance with previous reports [66,67], thus confirming that neither TA- nor DN-p73 are essential to maintain ESC self-renewal capacity under unstressed proliferating culture conditions. However, despite this lack of effect on cell proliferation, differential gene expression analysis revealed some subtle, but significant, differences in the expression of several cell cycle-related genes (Figure 5C). A strong downregulation of the bona fide TAp73 transcriptional target *Cdnk1a* (p21) [68] was observed, validating, once more, that the generated cell lines are good p73 isoform-specific knockout models. In addition, our RNA-seq data revealed a significant upregulation of *Ccnd2* (Cyclin D2), that was also confirmed by qRT-PCR, in both TA- and DN-p73KO cell lines (Figure 5C,D). These results agree with previous reports in cycling somatic cells, in which TAp73 and DNp73 support cellular proliferation by activating several cell cycle progression genes, including *CCND3* (Cyclin D3), through binding to the p53-responsive elements present in their regulatory regions [69].

Compiled evidence indicates that core cell cycle mechanisms operate differently in ESC than in somatic cells [70,71,72]. Thus, TAp73 and DNp73 regulation of the G1 phase machinery in these cells was particularly interesting. mESC cells express low levels of D-type cyclins, and their unique cell cycle organization has a link to the maintenance of pluripotency [71]. It has been proposed that in ESCs the G1 phase may enable the accumulation of factors needed for the dissolution of pluripotency and differentiation [72]. Upregulation of CyclinD-Cdk4/6 kinases during the late G1 phase triggers the phosphorylation and cytoplasmic retention of Smad2/3, preventing endodermal cell fate and rendering cells susceptible only to neuroectodermal differentiation [73]. The detected upregulation of *Cdk6* expression by RNA-seq was further confirmed by qRT-PCR in both cell lines (Figure 5C,E respectively). Intriguingly, cell cycle regulators are also necessary for mesoderm formation [74], again linking cell cycle machinery to pluripotency exit and germ layer specification. p73 appears to be essential to maintain the appropriate transcriptional profile of the G1 phase, a permissive phase for the initiation of cell fate decisions. Thus, p73 might work as a switch in the G1 phase to control early cell fate decisions and pluripotency.

### 3.5. Inactivation of p73 Isoforms Is Compatible with mESC Pluripotency, but It Predisposes Naïve Pluripotent mESC to Transition into Primed Cells, and Compromises Adequate Lineage Differentiation in an Isoform-Specific Manner

**Figure 6:**
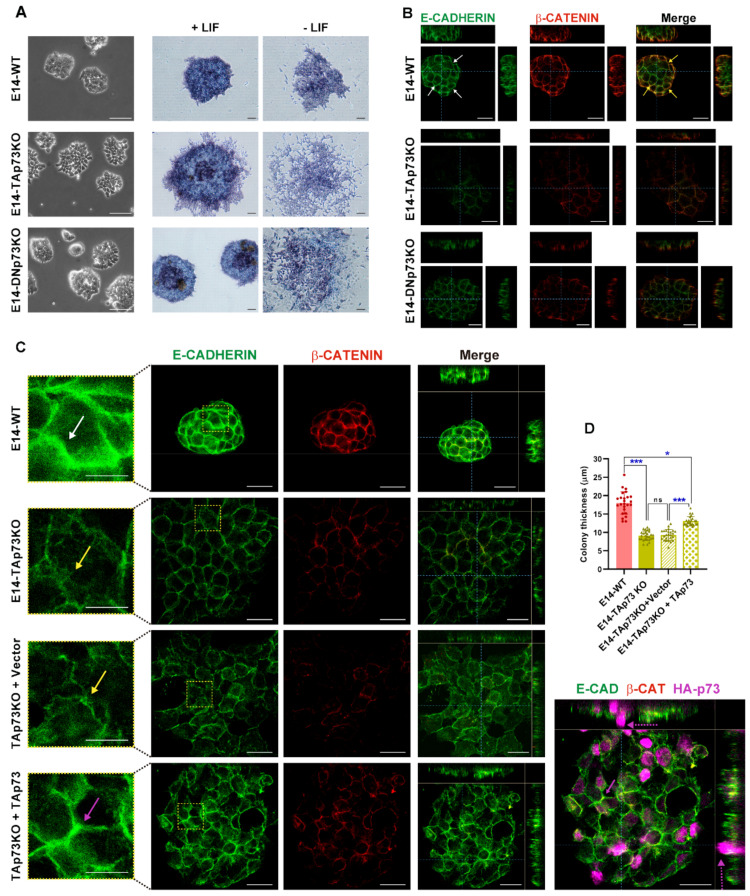
TAp73 is required for the establishment of appropriate adherens junctions (AJs) and its absence results in the formation of mESC colonies with a “primed” phenotype. (**A**) Representative images showing characteristic colony morphology of WT and knockout E14 cells seeded under stemness conditions and images of alkaline phosphatase activity for colonies grown in the presence or absence of LIF. Scale bar: 100 μm. (**B**,**C**) Analysis of AJs and colony thickness by confocal microscopy orthogonal projections of three-dimensional reconstruction images in the indicated genotypes (**B**) or after TAp73 ectopic expression in E14-TAp73KO cells (**C**). Cells were immunostained with anti-β-catenin (red) and anti-E-cadherin (green) antibodies. Scale bar: 20 μm. White arrows indicate continuous and tight AJ (**B**,**C**), yellow arrows pleated and broken up AJ (**C**) and pink and dotted arrows indicate recovered AJ and second layer of cells after TAp73KO re-expression, respectively. The insert represents a magnification of the indicated area (RED square). (**D**) Quantification of colony thickness using orthogonal projections from Z-stacks of confocal microscopy images. * *p*-value < 0.05, *** *p*-value < 0.001.

Depending on their state of pluripotency, mESC colonies adopt different morphological appearances. When cultured under proliferating conditions (S/L), mESC in naïve state form domed colonies, while cells in primed state establish flat colonies that resemble those of epiblast-derived stem cells (EpiSCs) [75]. Under such conditions, E14-WT cells formed the characteristic naïve round and tightly packed domed colonies. These colonies showed alkaline phosphatase (AP) activity, which soon diminished in media without LIF (Figure 6A). Strikingly, all TAp73KO cell lines formed irregular colonies with loosely attached cells that gave them a less compact and flat appearance, despite the stemness conditions in which they were cultured (Figure 6A and Supplementary Figure S3A). Meanwhile, most E14-DNp73KO colonies looked similar to E14-WT, but some had cells migrating away from the colony, such as the TAp73KOs (Figure 6A and Supplementary Figure S3B). In both cases, upon LIF withdrawal, TA- and DN-p73KO cells rapidly changed their morphology and spread away from the core of the colony (Figure 6A).

E-cadherin expression and the establishment of adherens junctions (AJs) are crucial for colony compaction and full pluripotency maintenance [64,76]. In accordance, E14-WT cells express high levels of E-cadherin and tight and continuous AJs with E-cadherin being neatly localized at the plasma membrane (in green) and sharply costaining with β-catenin (in red) (Figure 6B,C, white arrows and insert). Confirming our previous observation, the cells in TAp73KO colonies had very low β-catenin staining and interrupted E-cadherin, reminiscent of feeble AJs (Figure 6B,C, yellow arrows). In these cells E-cadherin staining delineated pleated and irregular membranes (Figure 6C, insert, yellow arrows). This phenotype was common to TAp73KO colonies from the three lines, which had an irregular shape and weaker AJs, while DNp73KOs colonies were round and had stronger AJs (Supplementary Figure S4A).

In addition, flattened ESC colony morphology correlated with a less naïve pluripotent state where flat colonies are a feature of primed pluripotency [75,77]. Thus, we analyzed the 3D multilayer architecture of the colonies by performing orthogonal projections from Z-stacks of confocal images to quantify colony thickness. E14-WT colonies were dome-shaped and consisted of several layers of cells with an average thickness of 22 μm. E14-TAp73KO colonies were flat, with only one cell layer and, congruently, significatively thinner (Figure 6B, Supplementary Figure S4A–C). On the other hand, E14-DNp73KO colonies had an intermediate thickness and not all the clones showed significant differences with the WTs (Figure 6B, Supplementary Figure S4B,C). It is of note, that all the E14-TAp73KO clones formed colonies that were significantly thinner than those of the DNp73KO. Moreover, the mean thickness of the TAp73KO cells was significantly smaller than that observed for the DNp73KO genotype (Supplementary Figure S4B,C), indicating that lack of colony compaction is most likely the result of TAp73-deficiency.

Next, we sought to demonstrate that TAp73 was required for AJ establishment and ESC colony compaction and rule out the possibility that the phenotype observed was attributable to clonal effects. To that end, we attempted to restore the original phenotype by ectopically re-expressing TAp73 into the E14-TAp73KO cells. As depicted in Figure 6C, expression of TAp73 resulted in stronger AJs (pink arrow), restored the round shape of the colonies and partially restored the thickness of the colonies. The differences in colony thickness of the TAp73-transfected cells were highly significantly compared with the vector-transfected TAp73KO colonies, but still thinner than the WTs (Figure 6D). Orthogonal projection of TAp73KO colonies with TAp73 expression revealed that some of the transfected cells, with stronger AJs (pink arrow), were starting to form a second tier of cells (Figure 6C, dotted pink arrow). Altogether, the data strongly demonstrate that TAp73 deficiency results in defects in the establishment of appropriate AJs, severely affecting the compaction of the ESC colonies, a phenotype that could reflect a lack of a full pluripotent state in these mutant cells and a predisposition to differentiation.

**Figure 7:**
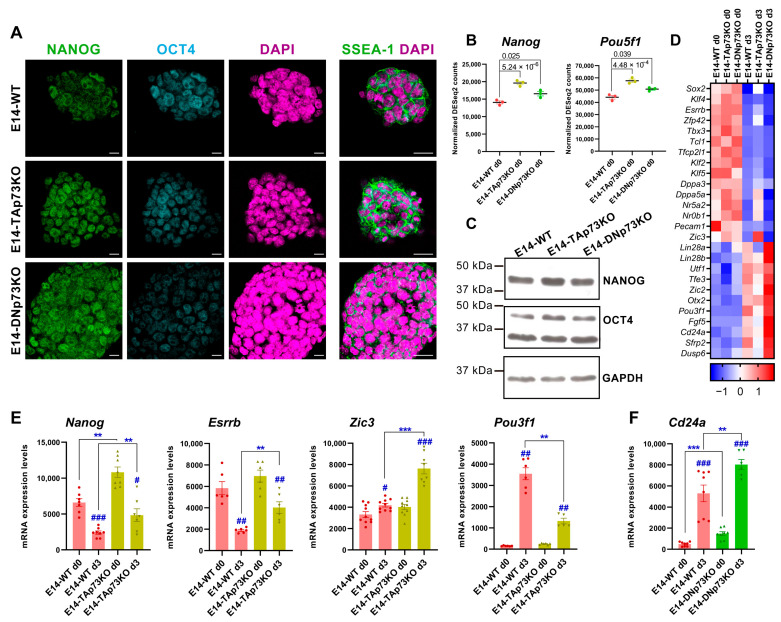
Lack of p73 isoforms predisposed naïve pluripotent mESC to transition into primed cells in an isoform-specific manner. (**A**) Confocal microscopy analysis of pluripotency markers NANOG (green), OCT4 (blue) and SSEA-1 (green). DAPI (purple) was used to visualize nuclei. Scale bars: 20 μm. (**B**) Expression plots showing normalized DEseq2 counts for *Nanog* and *Pou5f1* from E14-WT and p73-isoform-specific KO samples under basal conditions (S/L, day 0). p-adj values are indicated. (**C**) Western blot analysis of NANOG and OCT4 protein levels in E14-WT and p73 isoform-specific KO cells. GADPH was used as loading control. (**D**) Heatmap visualization of RNA-Seq expression Z-scores for regulatory genes of core and naïve pluripotency, as well as markers of transition and primed pluripotency in E14-WT and p73 isoform-specific KO samples from day 0 and day 3. Average values for each condition are plotted. (**E**,**F**) Analysis of pluripotency markers by qRT-PCR in E14-TAp73KO (**E**) or E14-DNp73KO (**F**) cells. # *p*-value < 0.05, **, ## *p*-value < 0.01, ***, ### *p*-value < 0.001.

The parental E14-WT cells, which are representatives of cells in naïve pluripotency state, exhibit distinct transcriptional profiles when maintained under stemness culture conditions (S/L) [75]. Thus, we asked whether the elimination of each individual p73 isoform affected such state. E14-WT and all the p73-isoform-specific knockout mESC lines expressed key molecular pluripotency markers like OCT4 and NANOG, which play essential roles in maintaining stemness as well as the cell-surface antigens SSEA-1 (Stage-Specific Embryonic Antigen-1) [78,79] (Figure 7A and Supplementary Figure S5). Thus, specific inactivation of p73 isoforms is compatible with mESC stemness. However, we detected slight differences in the expression levels of *Nanog* (NANOG) and *Pou5f1* (OCT4), especially in TAp73KO cells compared to E14-WT control (Figure 7B,C,E). To further address if the elimination of p73 isoforms could alter transcriptional profiles associated to pluripotency, we used our RNA-seq data from the cells grown under S/L conditions to compare the expression levels of regulatory genes of core and naïve pluripotency, as well as transition and primed pluripotency markers (Figure 7D, day 0). Our data revealed that among the DEGs in E14-TAp73KO cells there was a significant upregulation of a group of transcription factors involved in supporting naïve pluripotency, such as *Nanog*, *Tfcp2l1* or *Tbx3* (Figure 7D). On the other hand, stemness maintenance genes such as *Lin28*, *Pecam1* or *Utf1* were downregulated DEGs in the absence of TAp73 (Figure 7D), despite the stemness culture conditions, similarly to what happens in EpiSC [75]. This data supports the idea that, while elimination of TAp73 is compatible with pluripotency, it hinders the naïve state of the mESC.

On the other hand, the elimination of DNp73 appears to have a more subtle effect (Figure 7D). However, some of the genes that control the transition from naïve to primed pluripotency, such as *Lin28a* or *Lin28b*, were upregulated compared to E14-WT. Interestingly, even under S/L culture conditions, these cells significantly overexpressed the early neural marker *Tubb3* (βIII tubulin, p-adj 0.03, log2FC 0.51) suggesting the premature differentiation of these cells. In this context, it is worthy of mention the exclusive *Klf5* downregulation in E14-DNp73KO cells, since *Klf5* is required to maintain ESCs in an undifferentiated state [80,81]. Moreover, *Klf5* downregulation in mESCs induces their spontaneous differentiation, as well as expression of various differentiation markers such as βIII tubulin, suggesting that downregulation of *Klf5* in E14-DNp73KO cells could predispose them to differentiate to an early differentiation and, in particular, to accelerate the kinetics of neural lineages [82,83].

To further address whether the lack of p73 isoforms affects the transition of naïve mESCs into primed cells, we analyzed the expression profiles of E14-TA- and E14-DN-p73KO cells after three days in culture under prodifferentiation conditions (EBs, serum without LIF). LIF removal causes mESC to exit their pluripotent state driven by autocrine signals and to progress from the naïve state to multi-lineage specification in an orderly sequence [84]. In agreement with these published data, naïve transcription factors such as *Esrrb* and *Tfcp2l1*, as well as *Zfp42* and *Klf2*, were strongly downregulated in WT-E14 cells after three days under EB culture condition compared to day 0. However, in the absence of TAp73, the above-mentioned genes, *Esrrb*, *Tfcp2l1* and *Zfp4,2* remained significantly upregulated after three days of differentiation, suggesting that TAp73 perturbs this transition (Figure 7D). Concurrently, expression of positive regulators of transition and early postimplantation epiblast markers such as *Otx2* and *Oct6* (*Pou3f1*) were upregulated in E14-WT cells at day 3 (Figure 7D), while in TAp73KO cells *Oct6* was severely downregulated compared to E14-WT levels (Figure 7D).

Since some of the observed changes in the pluripotency markers in the RNA-seq data were modest, we decided to confirm some of them by qRT-PCR. In the case of *Nanog*, its enhanced expression in TAp73KO cells was maintained after three days under differentiating conditions, while in WT cells the expression of this pluripotency gene had begun to decline (Figure 7E). Consistently, levels of *Esrrb* were also maintained in TAp73KO cells at that point (Figure 7E). It is noteworthy that, even though *Zic3* is upstream of a set of prodifferentiation transcriptional regulators and controls the transition from naive to primed pluripotency [85], at this early stage of differentiation we only detected a slight, but significant, increase of *Zic3* in WT-E14 cells by qRT-PCR. However, we detected a significant induction of this gene in E14-TAp73KO at day 3 (Figure 7E). Concomitantly, *Pou3f1* (Oct6) was severely downregulated in TAp73KO cells compared to levels in E14-WT (Figure 7E), altogether supporting the idea that TAp73-deficient cells might be predisposed to transition into primed mESC. Conversely, after three days under differentiation conditions, E14-DNp73KO cells strongly repressed the naïve transcription factors while upregulating, even more than E14-WT cells, the transition regulators and primed pluripotency markers (Figure 7D). The upregulation of *Cd24a*, which promotes neural precursor differentiation [86], governing the change of identity of ESCs and the commitment to differentiation [87] is of note. Upregulation of *Cd24a* was significant (p-adj 0.03, log2FC 1.38) as determined by RNA-seq analysis, and highly significant by qRT-PCR analysis, in E14-DNp73 cells (Figure 7F).

**Figure 8:**
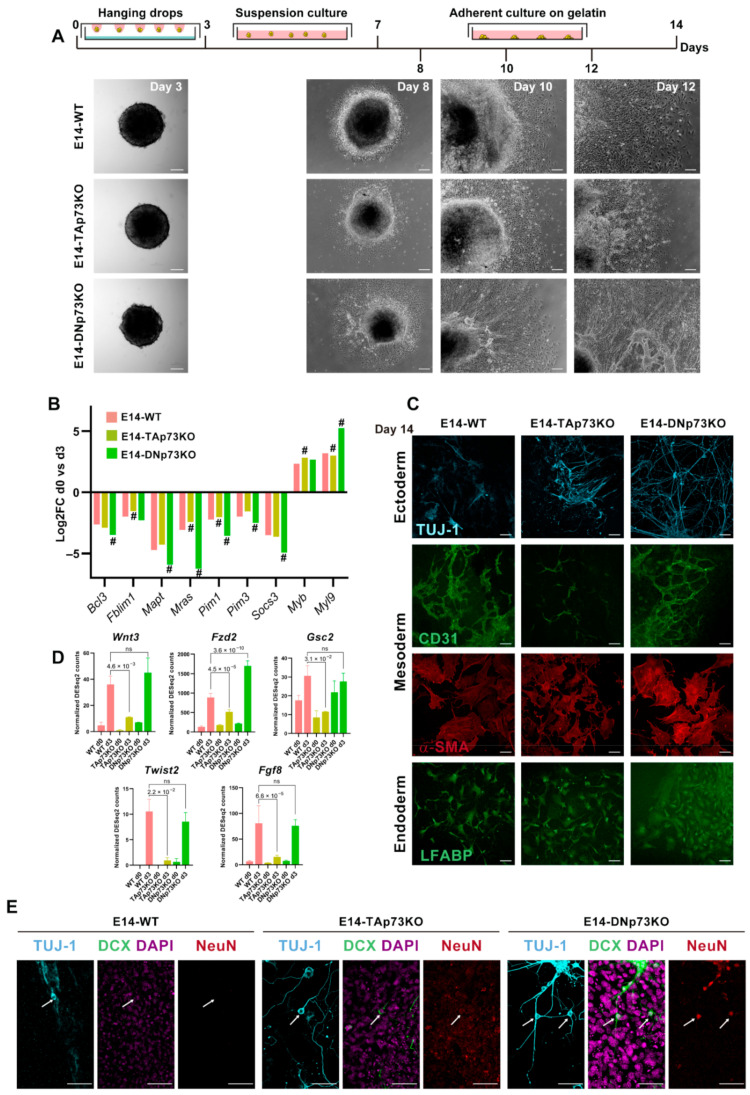
Inactivation of p73-isoforms compromises adequate lineage differentiation accelerating neural differentiation. (**A**) Schematic diagram of the spontaneous 2D-differentiation protocol and representative images of the EBs at different time points. Images were acquired by stereomicroscopy (day 3; scale bar: 100 μm) or phase-contrast microscopy (days 8-12; scale bar: 250 μm). (**B**) Fold-changes in expression of genes regulated during the early stages of the spontaneous differentiation process of mESCs, comparing day 0 with day 3. # Denotes statistically significant p-adj values. (**C**) Confocal microscopy images showing differentiation to the three germ layers: TUJ-1 (ectoderm, blue), CD31 and α-SMA (mesoderm, green and red, respectively) and LFABP (endoderm, green). Scale bar 50 μm. (**D**) RNA-seq analysis of mesodermal related genes showed a significant abatement in TAp73KO cells. Bar graphs depicts normalized DEseq2 counts. p-adj values are indicated. (**E**) Confocal microscopy images showing markers of neural differentiation: TUJ-1 (blue), Doublecortin (DCX, green) and NeuN (red). Nuclei were stained with DAPI (purple). White arrows show neuronal cell bodies. Scale bar 50 μm.

In vitro 2D and 3D differentiation models can be directed to specific cell types upon addition of specific chemicals or, on the contrary, spontaneous differentiation to the three germinal lineages can result from the withdrawal of LIF in the absence of any directing factors. Directed differentiation is determined by the exogenous chemicals used, but the spontaneous differentiation depends on the endogenous factors of the tested cells and the specific signaling pathways involved [88]. So far, our data supports the idea that elimination of the p73 isoforms might alter the kinetics of transition from naïve to primed mESCs in an isoform-specific manner. Thus, it is reasonable to hypothesize that specific elimination of p73 isoforms might affect the early stages of mESCs differentiation. To test this idea, we carried out a spontaneous differentiation protocol, as depicted in Figure 8A. We generated EBs in the absence of LIF and, after four days in suspension culture, EBs were transferred to gelatin-coated plates. All the cell lines showed efficient EB formation, but early on in the process we began to detect differences among them (Figure 8A). At day 8, the E14-WT cells had begun to migrate away from the EB. Surprisingly, at this point E14-DNp73KO EBs were already surrounded by a wide area of epithelial-like cells, comparable to what E14-WT EBs would attain by day 10. After 12 days in differentiation conditions, E14-DNp73KO EBs, and to a lesser extend E14-TAp73KO EBs, displayed tube-like structures that radiated from the cortex of the EB and connected and joined with each other forming thick networks, indicating differences in the kinetics of spontaneous differentiation.

Previous studies have documented some of the changes in gene expression during the early stages of the spontaneous differentiation process of mESCs [89]. These studies identified a group of signaling and cytoskeleton related factors as well as nuclear proteins (*Pim1*, *Pim3*, *Socs3*, *Anxa3*, *Mras*, *Fblim1*, *Vim*, *Mapt*, *Bcl3*, *Klf4*, *Klf5*, and *Nr0b1*) that were commonly downregulated in all the cell lines analyzed. Meanwhile, another set of genes, which included *Wdr1*, *Arpc5*, and *Myl9 Smn1*, *Myb*, and *Otx2*, were upregulated [89]. RNA-seq data indicated that these genes showed the same tendency of regulation after three days in differentiating conditions in the cell from the three genotypes. However, p73 isoform-specific knockout cells displayed significant differences in the magnitudes of this regulation compared to E14-WT (Figure 8B). In E14-DNp73KO cells the up and downregulation of these genes was significantly stronger than in E14-WT cells, suggesting that either the differentiation process was accelerated, or it had stronger signaling. Conversely, this regulation was weaker in E14-TAp73KO cells compared to E14-WT, highlighting possible deficiencies in the differentiation process. Thus, specific elimination of TA- and DN-p73 isoforms affects the transcriptional programs of the early stages of the differentiation process in an isoform-specific manner.

The EBs generated from mESCs are known to resemble the anterior prestreak stage of the embryo development with the epiblast-like core capable of generating derivatives of all three primary germ layers: definitive endoderm, mesoderm, and ectoderm [90]. In accordance, immunostaining of the EBs from all the cell lines after 14 days of differentiation showed positive staining for lineage markers of three germ layers (Figure 8C): βIII-tubulin, Tuj-1 (ectoderm), liver fatty acid-binding protein (LFABP; endoderm), and platelet endothelial cell adhesion molecule and α-smooth muscle actin (CD31 and αSMA, respectively, mesoderm), demonstrating that the knockout cell lines had a certain level of pluripotency.

Regarding mesodermal specification, E14-WT and E14-DNp73KO cells showed strong CD31 and SMA staining, and both cultures developed CD31-positive tubular networks, typical of endothelial differentiation (Figure 7C). Conversely, although TAp73-deficient cells expressed both markers, they failed to form vascular networks (Figure 8C). This result confirms previous reports that discovered p73 as an essential factor for vascular morphogenesis [5]. Interestingly, RNA-seq analysis of E14-WT and E14-TAp73KO cells after three days of differentiation revealed significant downregulation of some mesodermal related genes such as *Wnt3*, *Fzd2*, *Gsc2*, *Twist2* and *Fgf8* (Figure 8D). This agrees with a previous study using double and triple knockouts of p53 family members in mouse and human ESC that indicated that p53 family redundantly drives mesodermal specification during exit from pluripotency, with this signaling being critical for activation of mesoderm differentiation [2].

Neural induction during embryogenesis is one of the earliest events in specification of embryonic lineages, and formation of neuroectodermal cells is commonly observed in spontaneous differentiation of ESCs [91,92]. In accordance, we detected Tuj1-positive cells, a microtubular protein expressed in neural progenitor and immature postmitotic neurons, in all cultures after 14 days of differentiation (Figure 8C,E). At this stage, E14-WT-Tuj1-positive cells had mostly unipolar morphology and did not express the neuronal microtubule-associated protein Doublecortin (DCX), nor the mature neural marker NeuN. On the contrary, E14-TAp73KO cultures displayed neurons showing bipolar morphology with long thick processes on one side and shorter neurite extension on the other that where positive for DCX but not for NeuN (Figure 8E, arrows), suggesting an accelerated rate of neural differentiation, compared to E14-WT cells. This is consistent with previous reports that indicated that lack of total p73 in neural progenitor cells results in premature neuronal differentiation [8,9], again validating the generated cell lines as bona fide p73 isoform-specific knockout models. Moreover, TAp73 is involved in the maintenance of neural stem/progenitor cell self-renewal and differentiation throughout the regulation of *SOX-2*, *Hey-2*, *TRIM32* and *Notch* [93]. Interestingly, even though DNp73 deficiency has not been associated with accelerated neuronal differentiation, the phenotype detected in the DNp73KO clones relates to their predisposition to premature neural differentiation. When cultured in the absence of LIF (spontaneous differentiation), but without any addition of specific chemicals to direct the differentiation to a specific lineage, E14-DNp73KO cultures displayed neurons with mature cell morphologies characterized by multiple, long, branching processes extending from the cell body and positive for the three analyzed markers, Tuj1/DCX/NeuN (Figure 8E, arrows). This striking premature differentiation was reproduced in all the E14-DNp73KO clones analyzed (Supplementary Figure S6), demonstrating that it is a phenotype caused by the elimination of DNp73 rather than a clonal effect. Overall, our data confirms that inactivation of p73 isoforms compromises adequate lineage differentiation in an isoform specific manner.

## 4. Conclusions

Altogether, we have generated and validated novel TA- and DNp73-deficient mESC cell lines that constitute physiological models for recapitulating in vitro the main steps of embryonic development, providing a useful tool for biomedical and developmental biology research. Due to the unique properties of ESCs, the generated lines could become a very useful resource to uncover new p73 functions that not only would impinge in the understanding of the role of p73 in cancer but could also go beyond its tumor suppressor function. Moreover, our data confirms that TAp73 deficiency results in defects in the establishment of appropriate AJs, severely affecting the compaction of the ESC colonies, a phenotype that could be linked to the lack of a full pluripotent state in the absence of TAp73. Indeed, the characterization of the generated cells indicated that the specific elimination of TA- or DN-p73 was compatible with mESC pluripotency but predisposed naïve pluripotent cells to transition into primed cells, compromising adequate lineage differentiation and suggesting that the differential expression of p73 isoforms might act as a rheostat during early cell fate determination.

## Data Availability

Supplementary Tables are available at https://open.scayle.es/dataset/lopez-ferreras-et-al-cancers-2021.

## Supplementary Materials

The following are available online at https://www.mdpi.com/article/10.3390/cancers13133182/s1, Figure S1: Confirmation of the correct gene editing of the E14-TA- and E14-DN-p73KO clones, Figure S2: Expression levels of the p53 family members by qRT-PCR analysis, Figure S3: mESC characteristic colony phenotype is lost in E14-TAp73KO cells, Figure S4: p73 deletion affects colony thickness, Figure S5: Expression of pluripotency markers, Figure S6: Neural premature differentiation is present in all E14-DNp73KO clones, Table S1: Genome-wide transcriptome profiling of the three genotypes under self-renewal or differentiation culture conditions, Table S2: List of differentially expressed genes which are direct or indirect transcriptional control of either TA- or DN-p73, Table S3: Comparison of differentially expressed genes in E14-TA- and E14-DNp73 cells with p73 transcriptional targets analyzed in Wang et al. (2020).
