# Supplementary material for: Deciphering the Nature of Trp73 Isoforms in Mouse Embryonic Stem Cell Models: Generation of Isoform-Specific Deficient Cell Lines Using the CRISPR/Cas9 Gene Editing System
Source: Cancers (Basel). 2021 Jun 25;13(13):3182. doi: 10.3390/cancers13133182 (PMC8268375; doi:10.3390/cancers13133182)
Supplement: Supplementary file 1 [file cancers-13-03182-s001.zip › cancers-1203884-supplementary-materials.pdf]

cancers

MDPI

Article

Deciphering the Nature of *Trp*73 Isoforms in Mouse Embryonic Stem Cell Models: Generation of Isoform-Specific Deficient Cell Lines Using the CRISPR/Cas9 Gene Editing System

Lorena López-Ferreras, Nicole Martínez-García, Laura Maeso-Alonso, Marta Martín-López, Ángela Díez-Matilla, Javier Villoch-Fernandez, Hugo Alonso-Olivares, Margarita M. Marques and Maria C. Marin

Supplementary Materials:

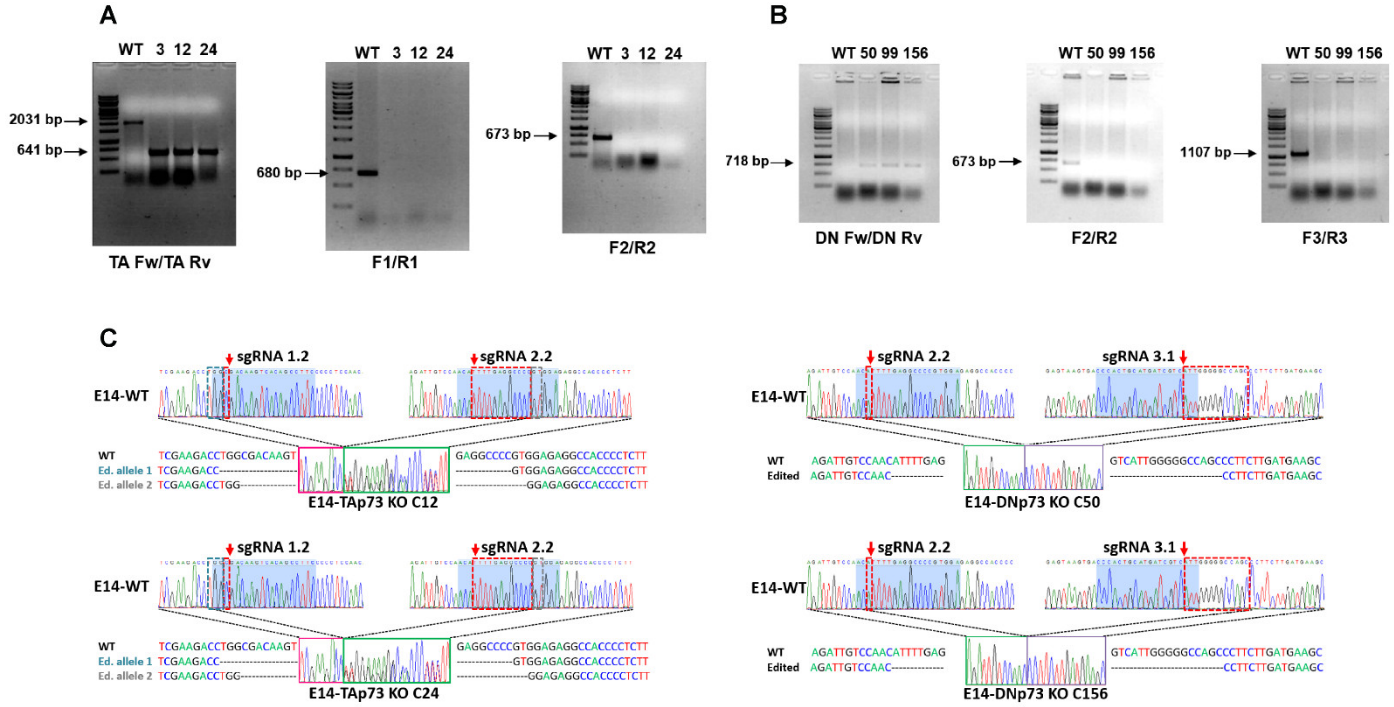

Figure S1. Confirmation of the correct gene editing of the E14-TA- and E14-DN-p73KO clones. (**A,B**) All E14-TA- (**A**) and E14-DN- (**B**) p73KO clones showed the expected PCR fragments (Primers Fw/Rv), while no amplicons, for amplification across the boundaries of the sgRNA binding regions (Primers F1/R1, F2/R2 and F3/R3), were detected. (**C**) Sequencing electropherograms from E14-WT cells and the selected clones. All the E14-TAp73KO clone show a 1390 bp deletion plus small different indels between both alleles in the boundaries of the deletion; while the E14-DNp73KO clones have a 7890 bp deletion (including a 14 bp indel, red dotted-squares), homozygous for both alleles. Blue-shadowed sequence corresponds to sgRNA target sequence.

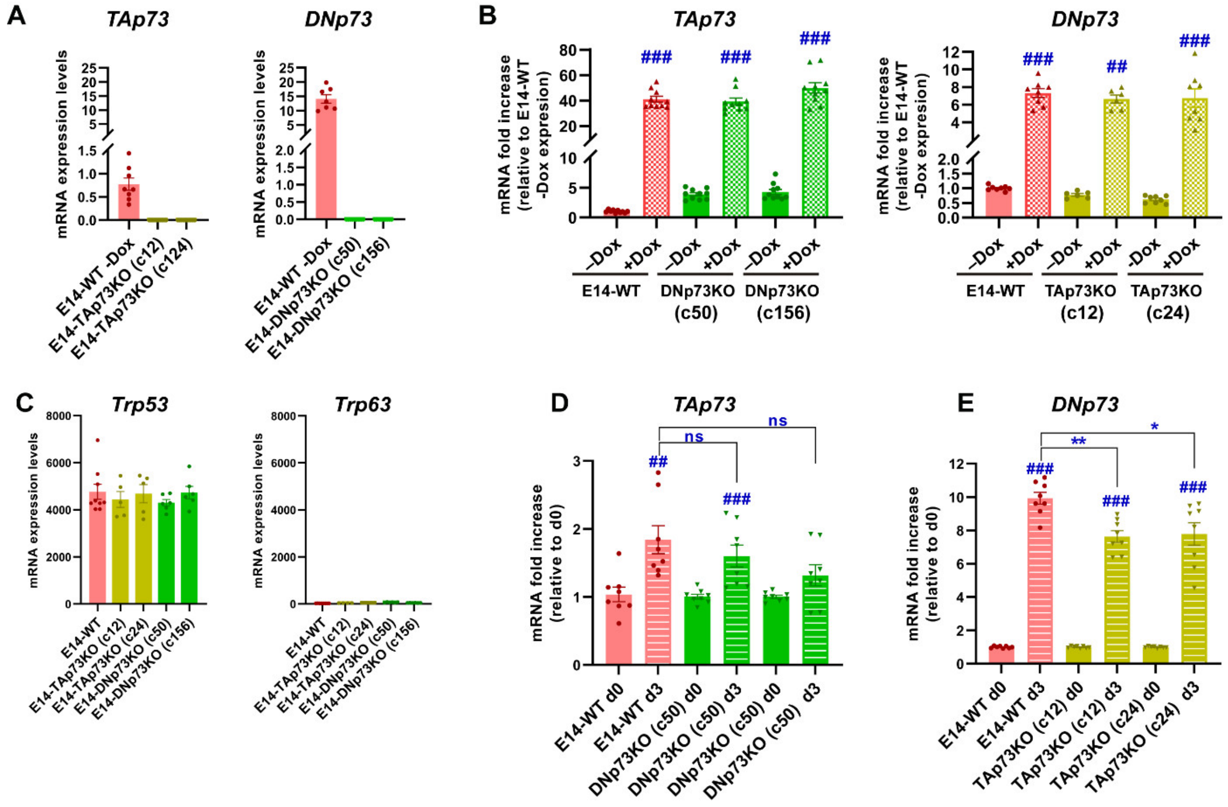

Figure S2. Expression levels of the p53 family members by qRT-PCR analysis. (**A**) Expression of the specific p73KO isoform under basal conditions. (**B**) TA- and DN-p73 expression upon treatment with doxorubicin (Dox). (**C**) Expression of *Trp53* and *Trp63*. (**D**,**E**) Expression of p73-isoforms under differentiation-permissive conditions (after 3 days of EBs culture, d3) compared to stemness basal conditions (d0, S/L) for DNp73KO (**D**) and TAp73KO (**E**) cells. The data represents the mean ± SEM of three independent experiments. * p-value < 0.05, **,## p-value < 0.01, **** p-value < 0.0001, ### p-value < 0.001.

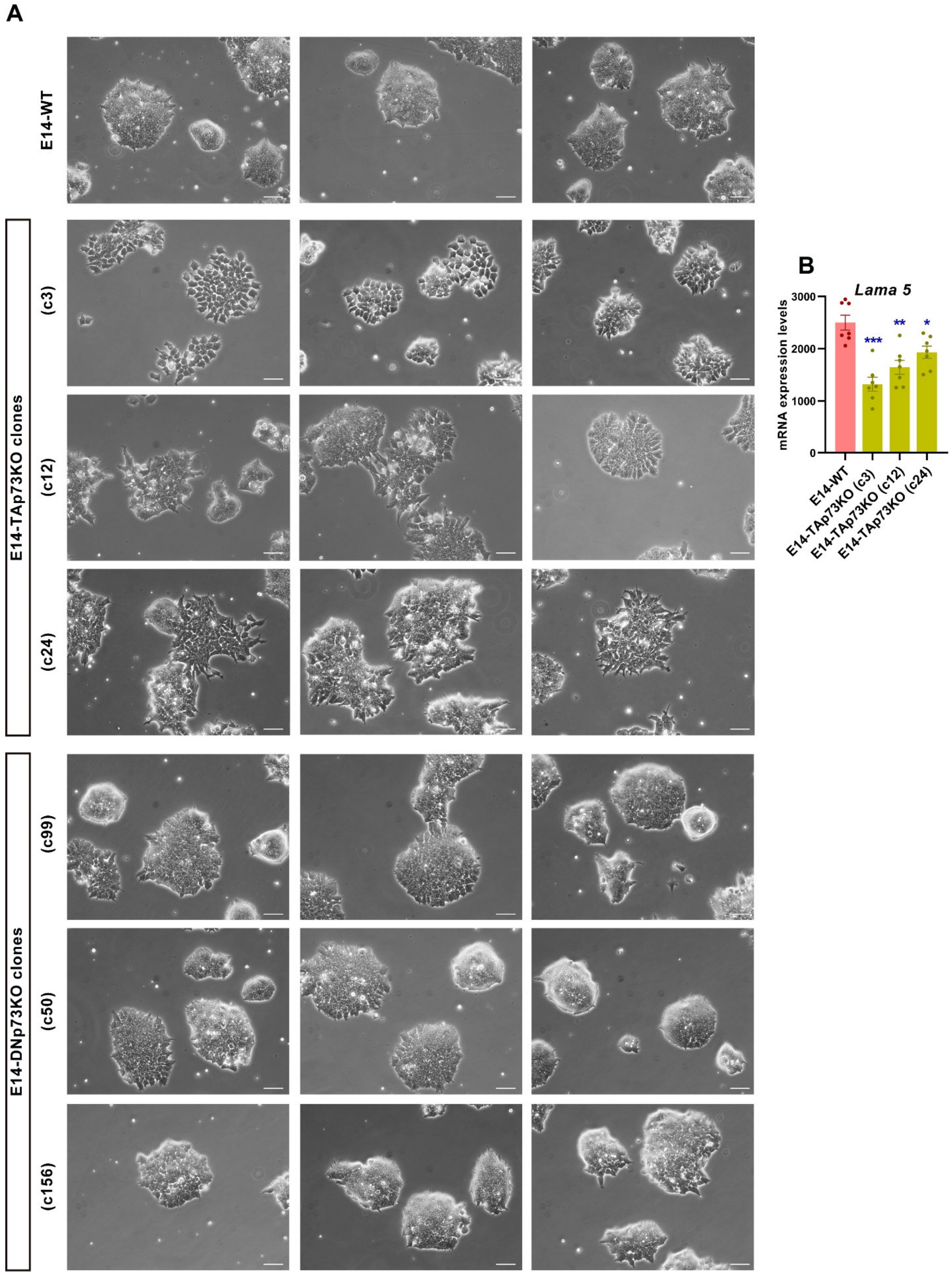

Figure S3. mESC characteristic colony phenotype is lost in E14-TAp73KO cells. (**A**) Representative images of the selected clones seeded at colony-forming density (2,500 cell/cm^2) under proliferating culture conditions (S/L). Scale bar: 75 µm. (**B**) Lama5 expression, analyzed by qRT-PCR, is lower in all the E14-TAp73KO clones compared to E14-WT cells. * p-value < 0.05, ** p-value < 0.01, *** p-value < 0.001.

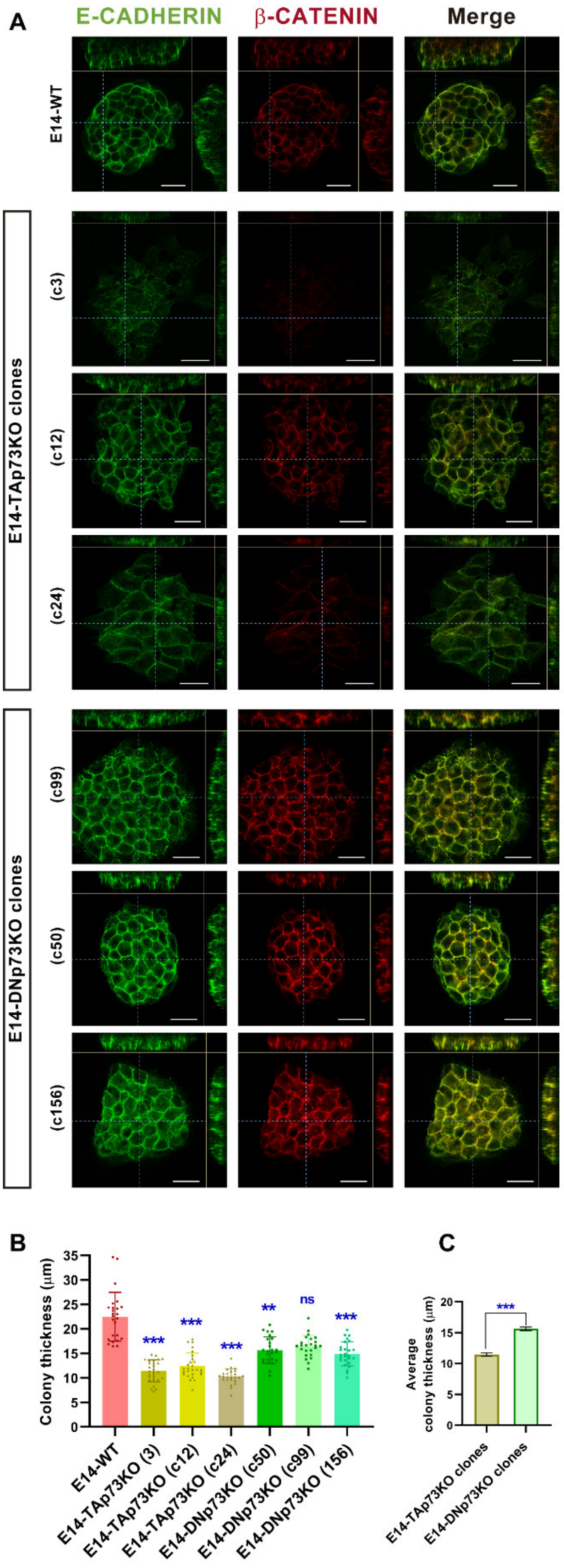

Figure S4. p73 deletion affects colony thickness. (**A**) Representative images of colonies of the different selected clones. Cells were immunostained with anti-E-cadherin (red) and anti-β-catenin (green) antibodies. Scale bar: 20 µm. (**B**) Quantification of the colony thickness compared to e14-WT cells by the orthogonal analysis of the figures. The data represents the mean ± SEM of at least the analysis of 10 independent colonies. (**C**) Comparison of the colony average thickness between E14-TA and E14-DN-p73Ko cells. ** p-value < 0.01, *** p-value < 0.001.

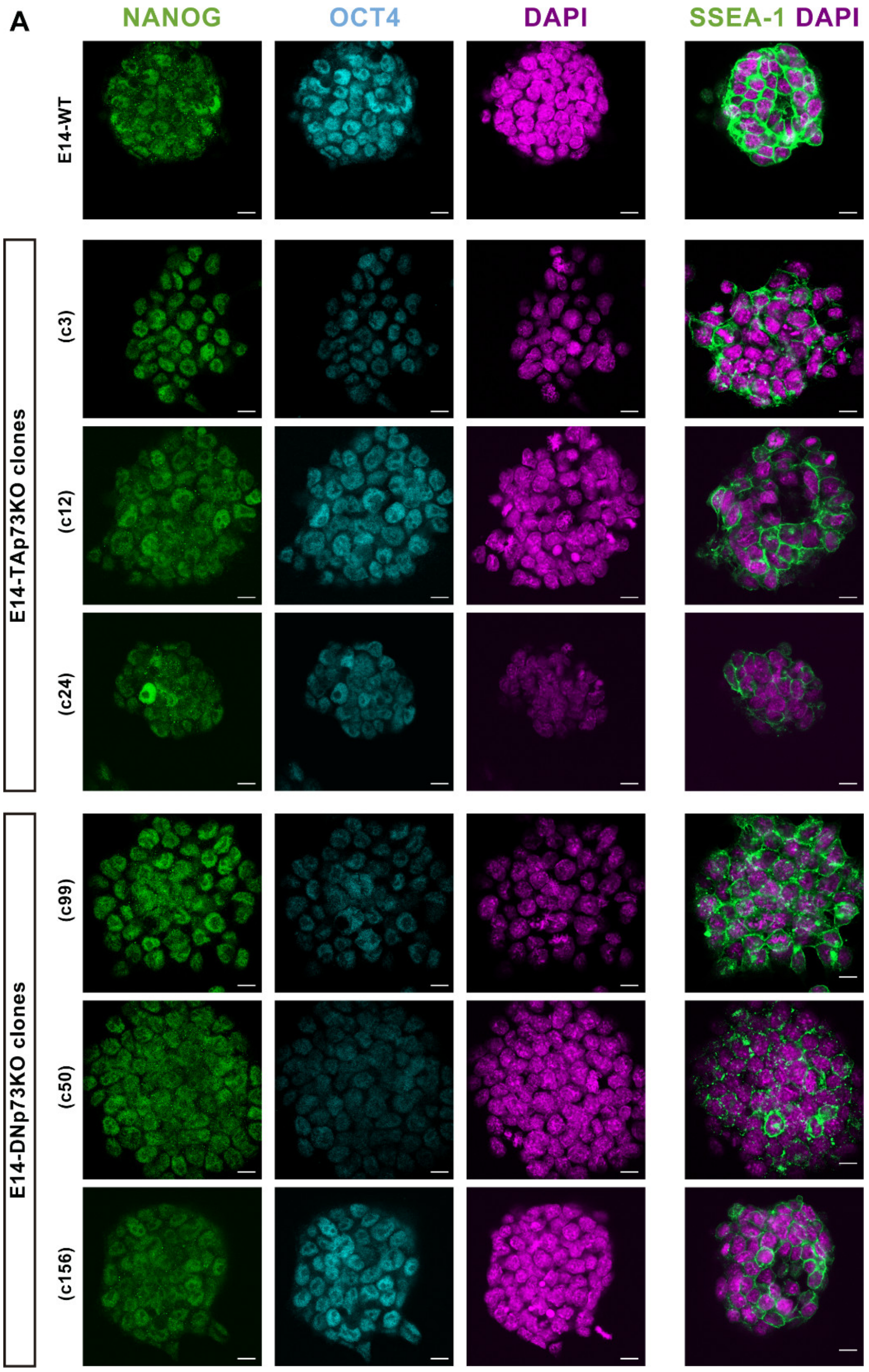

Figure S5. Expression of pluripotency markers. (**A**) Confocal microscopy analysis of pluripotency markers NANOG (green), OCT4 (blue) and SSEA-1 (green) in E14-TA- and E14-DN- p73KO clones. DAPI (purple) was used to visualize nuclei. Scale bars: 20 µm.

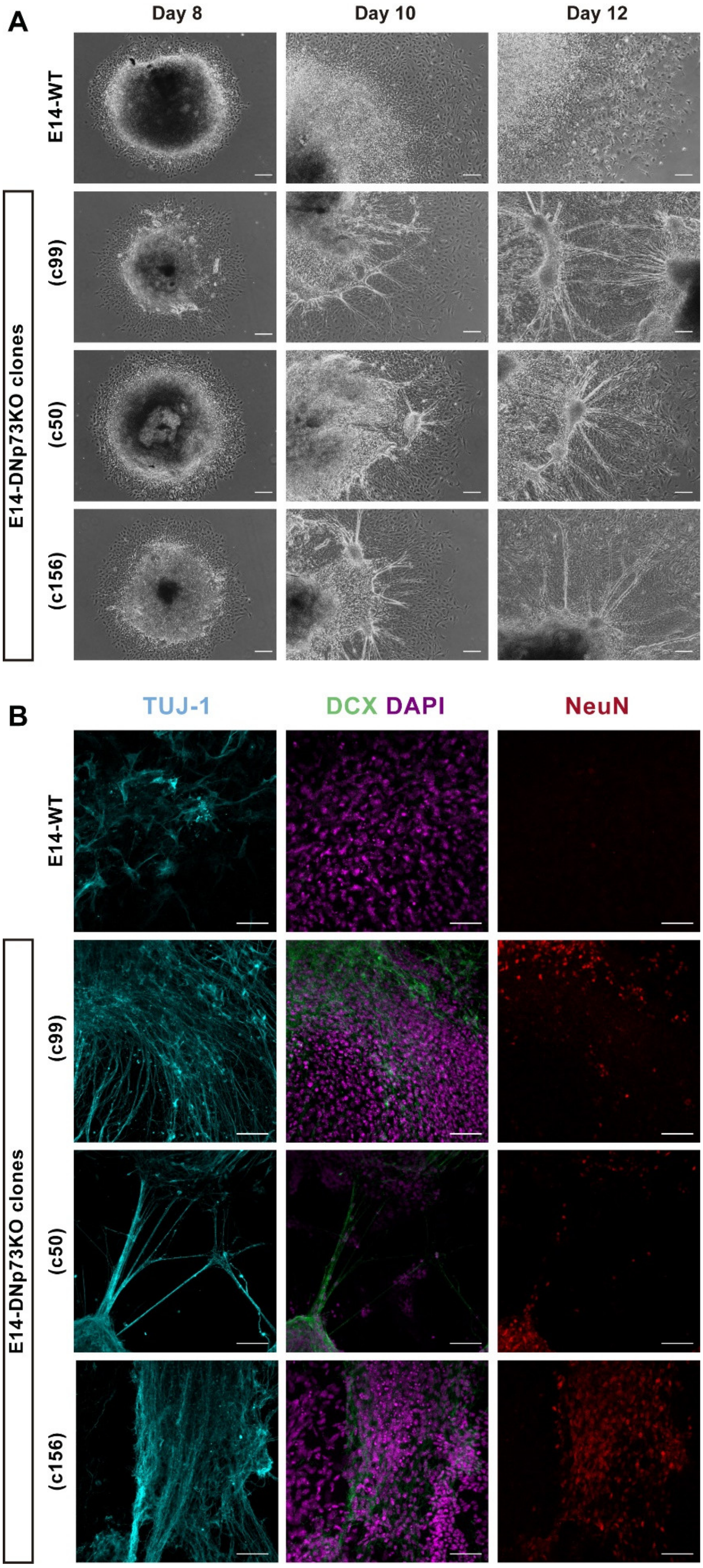

Figure S6. Neural premature differentiation is present in all E14-DNp73KO clones. (**A**) Representative phase-contrast microscopy images of embryonic bodies at different timepoints. (**B**) Confocal microscopy images showing markers of neural differentiation: TUJ-1 (blue), Double-cortin (DCX, green) and NeuN (red) after 14 days of differentiation. Nuclei were stained with DAPI (purple).
